# Supplementary material for: Expression of Oncolytic Adenovirus-Encoded RNAi Molecules Is Most Effective in a pri-miRNA Precursor Format
Source: Mol Ther Oncolytics. 2020 Oct 27;19:332–43. doi: 10.1016/j.omto.2020.10.012 (PMC7723779; doi:10.1016/j.omto.2020.10.012)
Supplement: Document S1. Tables S1–S3 and Figures S1–S5 [file mmc1.pdf]

OMTO, Volume *19*

Supplemental Information

Expression of Oncolytic Adenovirus-Encoded RNAi Molecules Is Most Effective in a pri-miRNA Precursor Format

Tereza Brachtlova, Jan-Willem van Ginkel, Mark J. Luinenburg, Renée X. de Menezes, Danijela Koppers-Lalic, D. Michiel Pegtel, Wenliang Dong, Tanja D. de Gruijl, and Victor W. van Beusechem

Supplemental Table 1: Synthetic DNA sequences encoding miRNA inserts prior to cloning into pSHAG-1. shmiR-1-2 and pre-miR-1-2 formats were obtained as two complementary oligonucleotides with overhangs compatible with BseRI/BamHI-digested pSHAG-1; pri-miR-1-1 and pri-miR-26b sequences were obtained as plasmid constructs with flanking restriction sites (coding sequence is given). Blue: passenger strand; Red: mature strand; **Bold**: RNA polymerase III transcription start nucleotide (G) and termination signal (TTTTT) .

Insert	DNA sequences of different microRNA formats
shmiR-1-2 coding	5’-**G**TTACATACTTCTTTACATTCAATAGAAGCTTGTATGGAATGTAAAGAAGTATGTAAC**TTTTT**-3’
shmiR-1-2 complementary	5’-GATCAAAAAGTTACATACTTCTTTACATTCCATACAAGCTTCTATTGAATGTAAAGAAGTATGTAACG-3’
pre-miR-1-2 coding	5’-**G**GTACATACTTCTTTATGTACCCATATGAACATACAATGCTATGGAATGTAAAGAAGTATGTATC**TTTTT**-3’
pre-miR-1-2 complementary	5’-GATCAAAAAGATACATACTTCTTTACATTCCATAGCATTGTATCTTCATATGGGTACATAAAGAAGTATGTACCG-3’
pri-miR-1-1	5’-*GAGGAG*GGACTCTACG**G**CAGTAGACTCCAGGGAAGAAGTTACACTGCCTCTGAGCTGCCTTCTCTACATCGCAGTGGGGTCAGCTTCTACCGGGGCGGCGTCCCGGGGTCTTGGAACTGCATGCAGACTGCCTGCTTGGGAAACATACTTCTTTATATGCCCATATGGACCTGCTAAGCTATGGAATGTAAAGAAGTATGTATCTCAGGCCGGGACCTCTCTCGCCGCACTGAGGGGCACTCCACACCACGGGGGCCGCCGCGGCCGACAGCCAGCCAGGCGGCTGTGGTGGGGGTGAGGCTGGGGCGGCAGGCTGCCTGGGCG**TTTTT***GGATCC*-3’
pri-miR-26b	5’-*GCAATG*CG**G**GTGTGAGACTTGTCCCAAAGTCACACAGAACCTCAAGGGCTTGTGCTGACTCCAAGCCTGCAGAGTGGGCTCCTCCTCTAGGCTCCCCCGTGCTGTGCTCCCTCGCCCCACCCTGCCCGGGACCCAGTTCAAGTAATTCAGGATAGGTTGTGTGCTGTCCAGCCTGTTCTCCATTACTTGGCTCGGGGACCGGTGCCCTGCAGCCTTGGGGTGAGGGGGCTGCCCCTGGATTCCTGCACTAGGCTGAGGTTGAGGCAGGGGAAGGGATTGGGAATTAGGGACCTCGTGAGGTAGGACTGGCCAGTGGAGTGGA**TTTTT***GGATCC*-3’

Supplemental Table 2: Virus titers determined as infectious units per ml (IU/ml).

Virus name	Production cell line	Determined titer (IU/ml)
AdΔ24E3-U6	A549	1.1 E+10
AdΔ24E3-U6.shmiR-1	A549	1.6 E+10
AdΔ24E3-U6.pre-miR-1	A549	1.4 E+10
AdΔ24E3-U6.pri-miR-1	A549	1.9 E+10
AdΔ24E3-U6.pri-miR-26b	A549	6.1 E+11
Long-term propagation AdΔ24E3-U6	HCT116	3.4 E+5
Long-term propagation AdΔ24E3-U6.primiR-1	HCT116	3.6 E+5

Supplemental Table 3: Primer sequences used for reverse transcription and qPCR.

Primer name	Primer sequence (5'→3')
PTGS2 forward	CCT GTG CCT GAT GAT TGC
PTGS2 reverse	CTG ATG CGT GAA GTG CTG
MET forward	AAA TAC GGT CCT ATG GCT GGT GGC
MET reverse	TGA AAT GGT TTG GGC TGG GG
FoxP1 forward	GCT TTT TAT GGC TGT GAG ACA CG
FoxP1 reverse	TTC TGG ATG GCT GAA CCG TTA C
U6 primer for RT	GTC ATC CTT GCG CAG G
U6 forward	CGC TTC GGC AGC ACA TAT AC
U6 reverse	AGG GGC CAT GCT AAT CTT CT
SL-miR-26b mature strand for RT	GTC GTA TCC AGT GCA GGG TCC GAG GTA TTC GCA CTG GAT ACG ACA CCT AT
miR-26b mature strand forward	GGG CCC GCT TCA AGT AAT TCA GGA T
SL-miR-26b passenger for RT	GTC GTA TCC AGT GCA GGG TCC GAG GTA TTC GCA CTG GAT ACG ACA GCC AA
miR-26b passenger strand forward	GCC CGC CCT GTT CTC CAT TAC TT
SL-universal reverse	CCA GTG CAG GGT CCG AGG TA
Packaging domain forward	GGA AGT GAC AAT TTT CGC GC
Packaging domain reverse	CCC GCG GCC CTA GAC AAA TAT

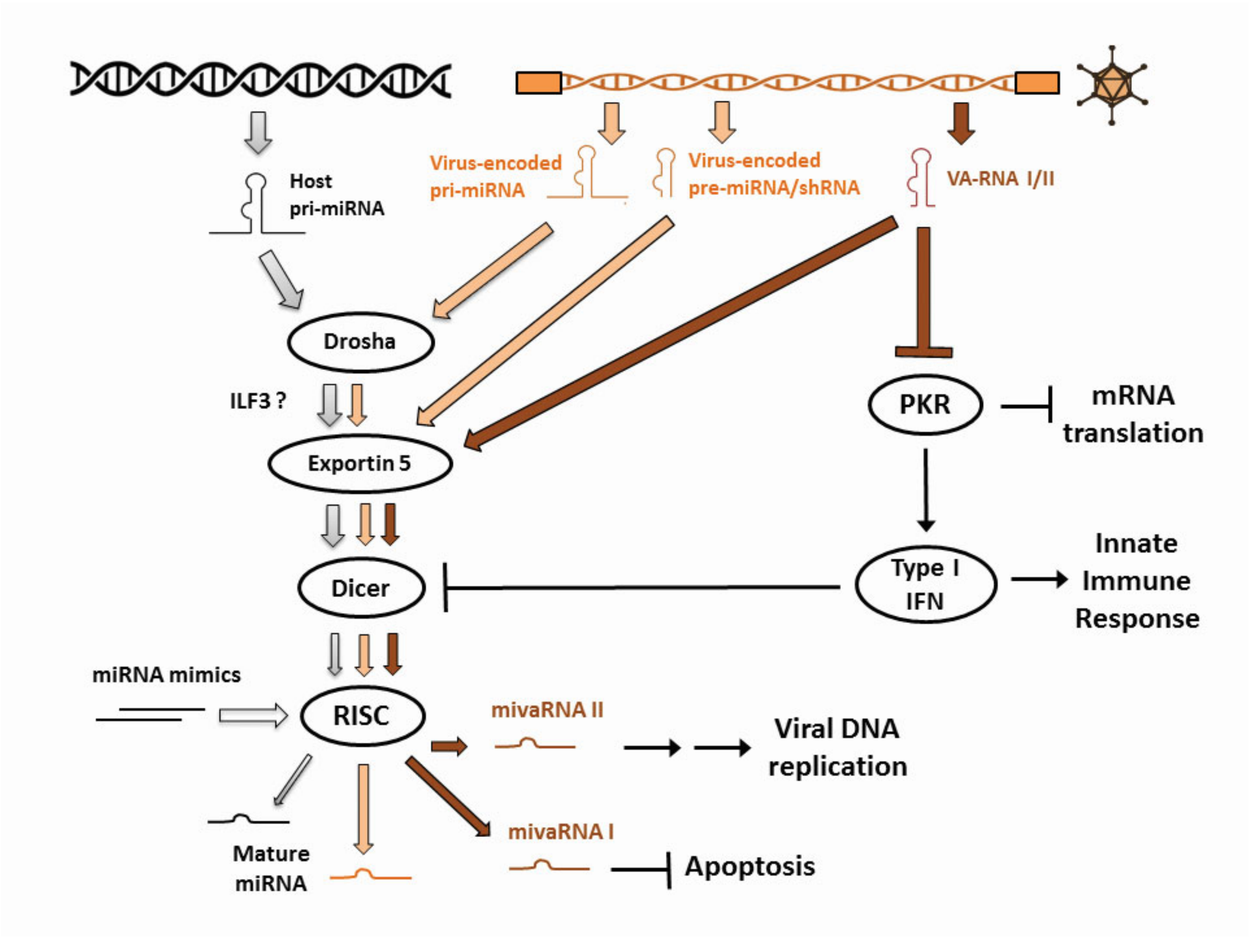

Supplemental Figure 1: Schematic microRNA processing of host-encoded endogenous miRNAs, adenovirus-associated RNAs and virus-encoded exogenous miRNAs.

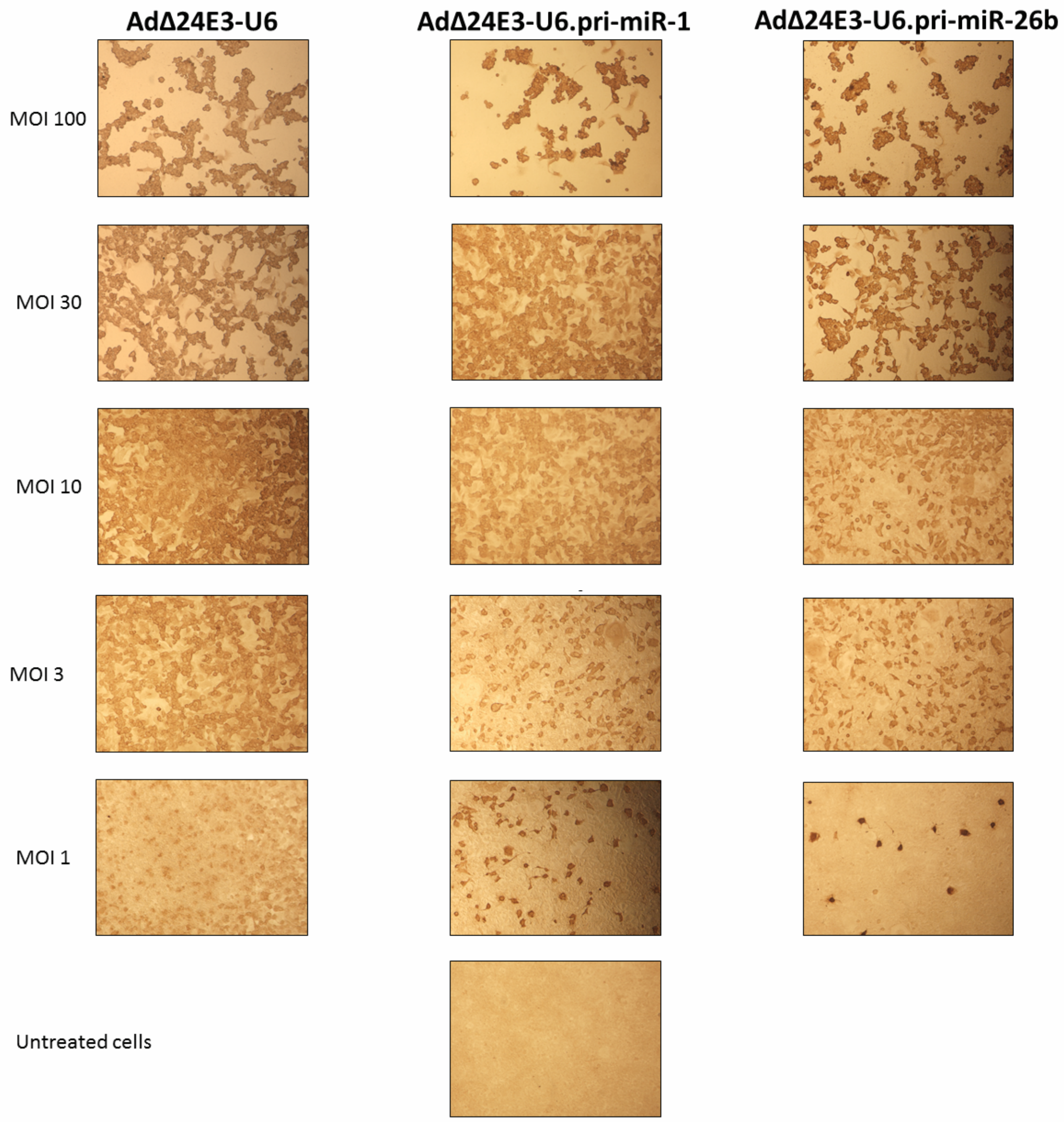

Supplemental Figure 2: Immunocytochemical staining of adenovirus hexon protein in 911 cells infected with AdΔ24E3-U6, AdΔ24E3-U6.pri-miR-1 or AdΔ24E3-U6.pri-miR-26b at various multiplicities of infection. At MOI-100, near complete infection is seen with all tested viruses.

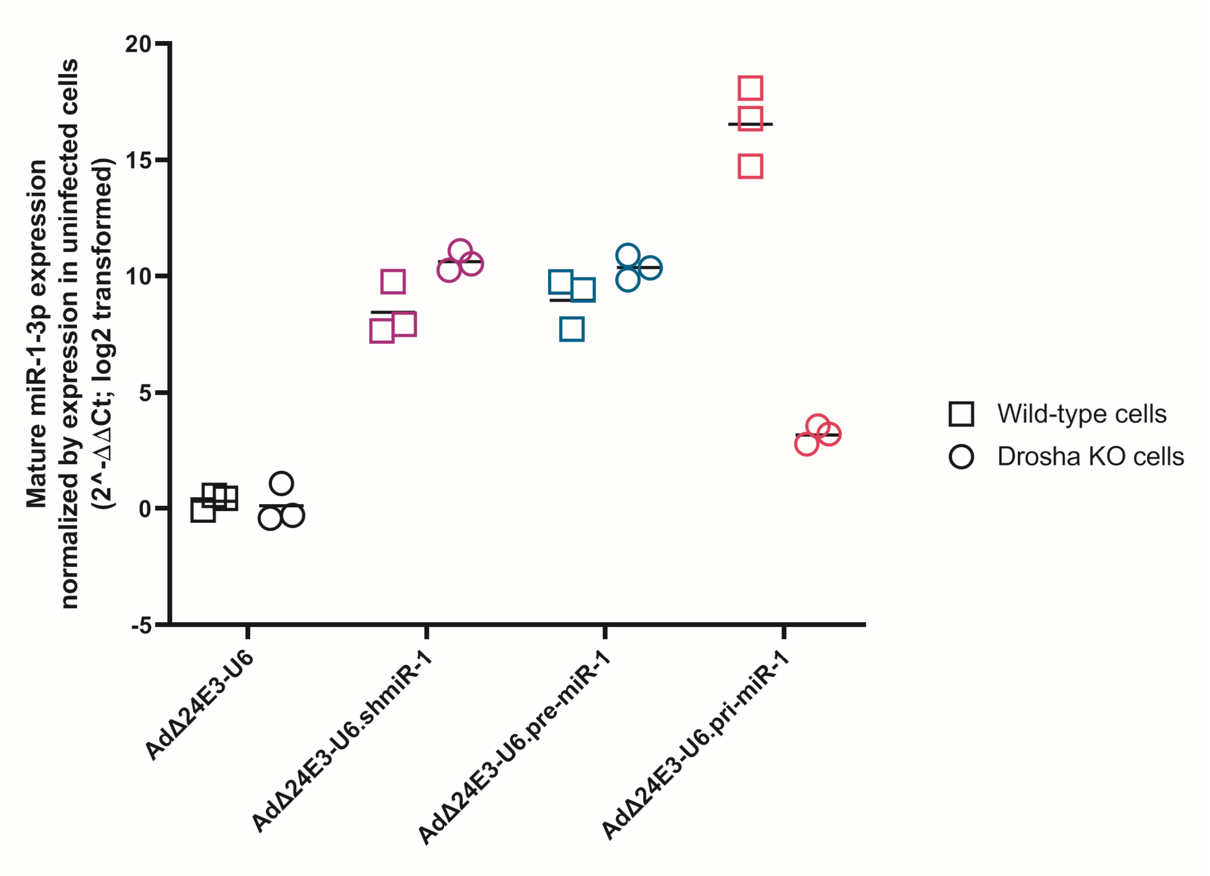

Supplemental Figure 3: The individual measurements in HCT116 wild type cells and *Drosha* knockout (KO) cells after log transformation for correction of skewed data distribution. Each dot represents the mean of one experiment (measured in technical duplicates). Three individual experiments were done.

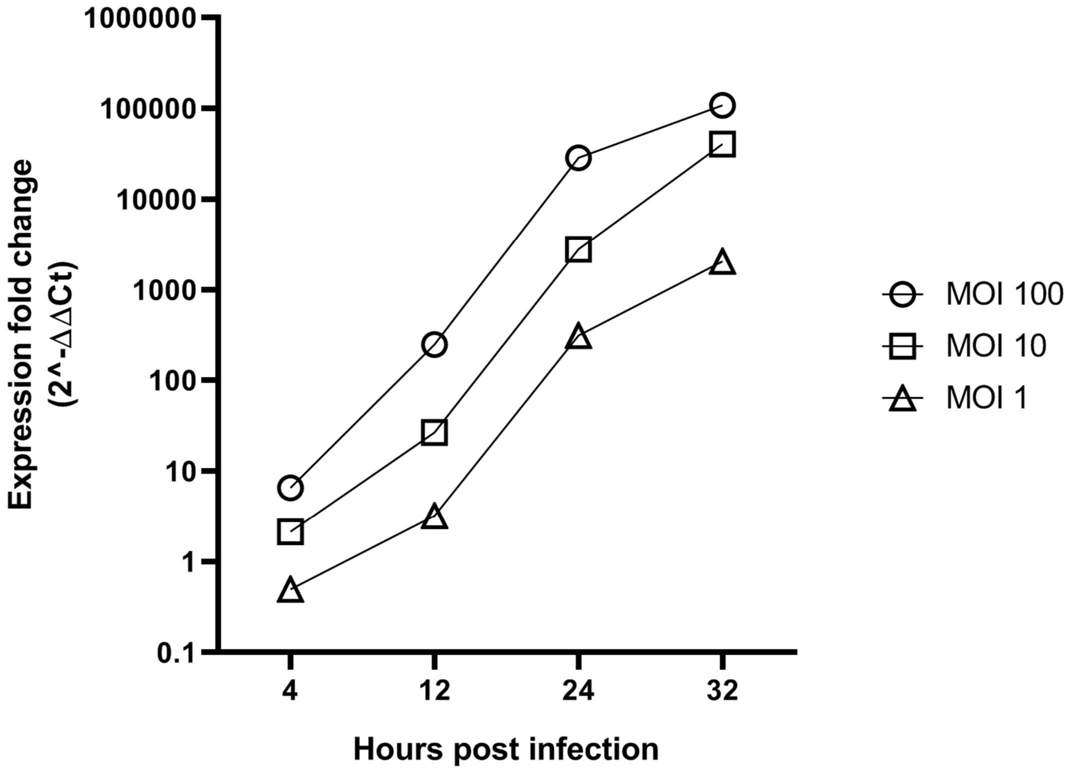

Supplemental Figure 4: Mature miR-1-3p levels in A549 cells infected with AdD24E3-U6.pri-miR-1 at the indicated MOI.

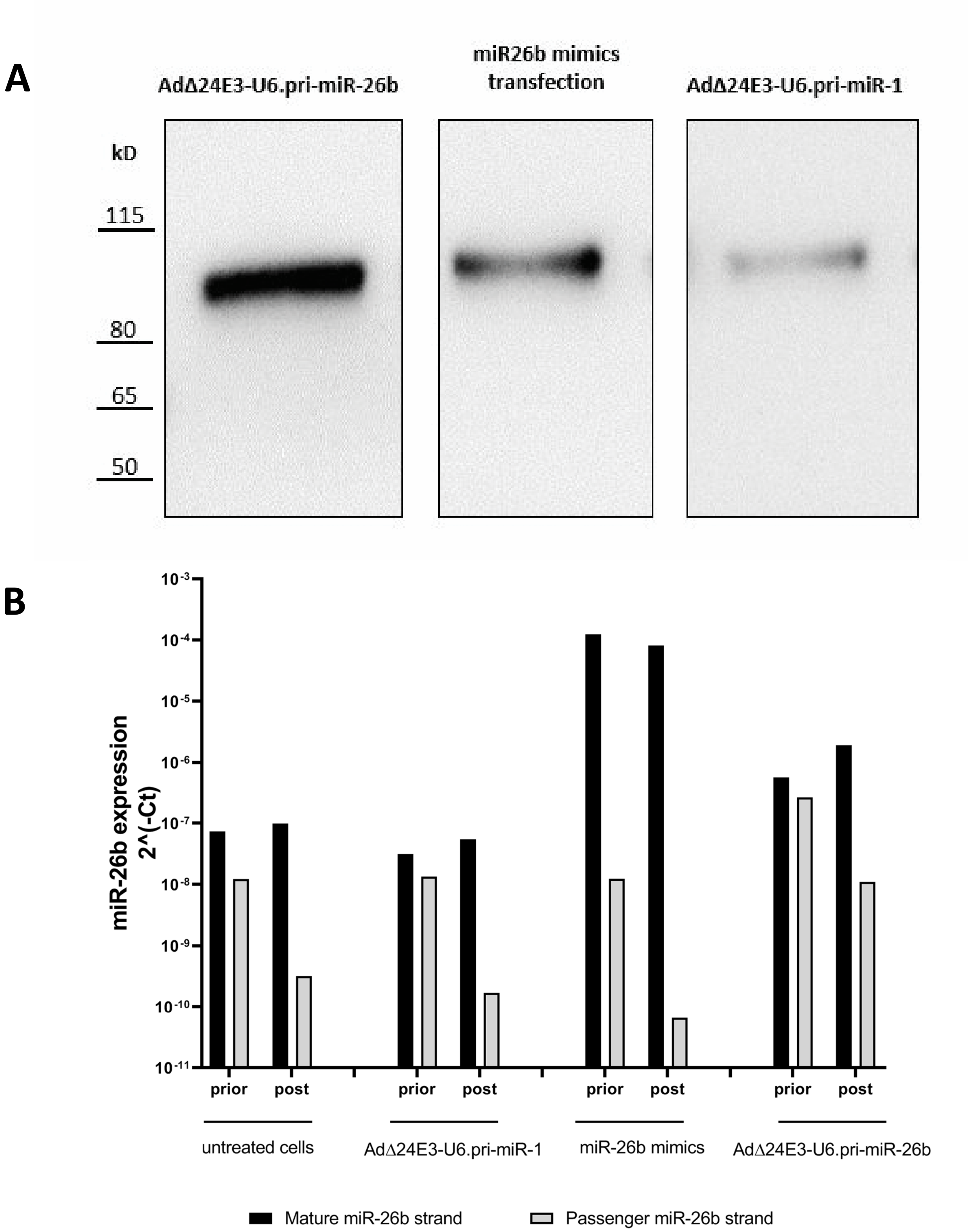

Supplemental Figure 5: Association of miR-26b mature and passenger strands to Argonaute RISC component (AGO) in HCT116 cells. (A) Pulldown of AGO from AdΔ24E3-U6.pri-miR-26b-infected cells, miR-26b mimics-transfected cells and AdΔ24E3-U6.pri-miR-1-infected cells. (B) Levels of mature miR-26b-5p and passenger miR-26b-3p in total cellular RNA (prior pulldown) and AGO-bound RNA (post pulldown). AGO-bound miR-26b was corrected for unspecific binding to beads (negligible). Data are normalized to the same amount input RNA prior to pulldown.
