# Supplementary material for: Expression of Oncolytic Adenovirus-Encoded RNAi Molecules Is Most Effective in a pri-miRNA Precursor Format
Source: Mol Ther Oncolytics. 2020 Oct 27;19:332–43. doi: 10.1016/j.omto.2020.10.012 (PMC7723779; doi:10.1016/j.omto.2020.10.012)
Supplement: Document S2. Article plus Supplemental Information [file mmc2.pdf]

Molecular Therapy
Oncolytics
Original Article

Expression of Oncolytic Adenovirus-Encoded RNAi Molecules Is Most Effective in a pri-miRNA Precursor Format

Tereza Brachtlova,[1,2] Jan-Willem van Ginkel,[2,6] Mark J. Luinenburg,[3,7] Renée X. de Menezes,[4,8] Danijela Koppers-Lalic,[3] D. Michiel Pegtel,[5] Wenliang Dong,[2] Tanja D. de Gruijl,[1] and Victor W. van Beusechem[1]

[1]Department of Medical Oncology, Amsterdam UMC, Vrije Universiteit Amsterdam, Cancer Center Amsterdam, Amsterdam Infection & Immunity Institute, De Boelelaan 1117, 1081 HV Amsterdam, the Netherlands; [2]ORCA Therapeutics B.V., 1081 HV Amsterdam, the Netherlands; [3]Department of Neurosurgery, Amsterdam UMC, Vrije Universiteit Amsterdam, Cancer Center Amsterdam, De Boelelaan 1117, 1081 HV Amsterdam, the Netherlands; [4]Department of Epidemiology and Biostatistics, Amsterdam UMC, Vrije Universiteit Amsterdam, Netherlands Bioinformatics Center, De Boelelaan 1117, 1081 HV Amsterdam, the Netherlands; [5]Department of Pathology, Amsterdam UMC, Vrije Universiteit Amsterdam, Cancer Center Amsterdam, De Boelelaan 1117, 1081 HV Amsterdam, the Netherlands

Oncolytic adenoviruses are being developed as new anti-cancer agents. Their efficacy can be improved by incorporating RNA interference (RNAi) molecules. RNAi molecules can be expressed in various precursor formats. The aim of this study was to determine the most effective format. To this end, we constructed three Δ24-type oncolytic adenoviruses, with human microRNA-1 (miR-1) expression cassettes in short hairpin RNA (shRNA), precursor microRNA (pre-miRNA), and primary miRNA (pri-miRNA) format, respectively. The viruses were compared for virus replication, mature miR-1 expression, and target gene silencing in cancer cells. Incorporation of the cassettes had only minor effects on virus replication. Mature miR-1 expression from the pri-miRNA format reached on average 100-fold higher levels than from the other two formats. This expression remained stable upon long-term virus propagation. Infection with the pri-miR-1-expressing virus silenced the validated miR-1 targets FOXP1 and MET. *Drosha* knockout almost completely abrogated mature miR-1 expression, confirming that processing of adenovirus-encoded pri-miR-1 was dependent on the host cell miRNA machinery. Using simple *in vitro* recombination cloning, a similar virus expressing miR-26b was made and shown to silence the validated miR-26b target PTGS2. We thus provide a platform for construction of oncolytic adenoviruses with high expression of RNAi molecules of choice.

INTRODUCTION

Although many advances were made in the treatment of cancer in recent years, cancer still remains one of the leading causes of death worldwide. There is, therefore, a great need for new treatments. One promising approach is oncolytic virus therapy, using viruses that selectively replicate in and kill cancer cells, evoking an antitumor immune response.[1,2] Several different oncolytic viruses are evaluated for this purpose, adenoviruses being among the most extensively studied. While preclinical results with these viruses are very promising, their anticancer efficacy as single agents in humans has generally been modest,[3] leaving room for improvement. Several approaches are investigated to accomplish this. One of the strategies is to use RNA interference (RNAi) technology. We previously showed that a short hairpin RNA (shRNA) can be expressed from the genome of an oncolytic adenovirus to silence a target gene in cancer cells.[4] This opened the way to develop more powerful oncolytic adenoviruses, by expressing selected shRNAs or microRNAs (miRNAs) during oncolytic virus replication in cancer cells (reviewed in Brachtlova et al.[5]). For example, oncolytic adenoviruses were made to silence VEGF to inhibit angiogenesis or an apoptosis inhibitor to promote cell death.[6,7] The results of these approaches were variable, which could be related to interactions between virus replication and RNAi biology, the choice of target genes, or the level of gene silencing that was achieved.[5]

The current study was initiated to assess an optimized format for high-level expression of RNAi molecules from the oncolytic adenovirus genome, to maximize gene silencing in infected cancer cells. Figure S1 illustrates the processing of endogenous host cell miRNAs and oncolytic adenovirus-encoded miRNA precursors in the RNAi machinery. Exogenous RNAi molecules can be expressed in different precursor formats that engage the cellular RNAi machinery at different levels.[8,9] To facilitate nuclear export and subsequent cytoplasmic processing into mature silencing duplexes, the RNA molecule

Received 11 March 2020; accepted 21 October 2020;
https://doi.org/10.1016/j.omto.2020.10.012.

[6]Present address: Kite Pharma EU B.V., Amsterdam, the Netherlands

[7]Present address: Amsterdam UMC, University of Amsterdam, (Neuro)Pathology, Meibergdreef 9, Amsterdam, the Netherlands

[8]Present address: Netherlands Cancer Institute, Biostatistics Unit, PSOE Department, P.O. Box 90203, Amsterdam, the Netherlands

Correspondence: Victor W. van Beusechem, Department of Medical Oncology, Amsterdam UMC, Vrije Universiteit Amsterdam, Cancer Center Amsterdam, Amsterdam Infection & Immunity Institute, De Boelelaan 1117, Room CCA 3.50, P.O. Box 7057, 1007 MB Amsterdam, the Netherlands.
E-mail: vw.vanbeusechem@amsterdamumc.nl

 © 2020 The Author(s).
This is an open access article under the CC BY license (http://creativecommons.org/licenses/by/4.0/).

should be transcribed and folded in at least the short hairpin-like structure that can be recognized by exportin 5 and Dicer (i.e., as shRNA or precursor miRNA [pre-miRNA]). Alternatively, the RNA can be transcribed as a long primary miRNA (pri-miRNA) transcript with embedded stem-loop RNA structure. Prior to nuclear export, this pri-miRNA is processed by the endonuclease DROSHA to release the pre-miRNA. During replication in host cells, adenoviruses express virus-associated RNAs (VA-RNAs), which are processed by the RNAi machinery into viral miRNAs.[10] In cells infected with miRNA-expressing adenoviruses, endogenous miRNAs, exogenous miRNAs, and VA-RNAs may thus compete for the processing capacity of the miRNA biogenesis pathway (Figure S1).

pri-miRNAs and pre-miRNAs typically contain imperfect hairpin structures with one or more mismatches in the double-stranded stem. In contrast, designed shRNAs that are matured by the RNAi machinery into short interfering RNAs (siRNAs) or miRNA mimics usually have (near) perfect sequence complementarity in the stem. To investigate the effects of nuclear DROSHA processing and of stem sequence complementarity on the efficiency of adenovirus-encoded miRNA processing, we compared oncolytic adenoviruses expressing a human miRNA in shRNA, pre-miRNA, or pri-miRNA format for production of mature miRNA and gene silencing efficiency. In addition, we compared oncolytic replication and assessed stability of RNAi molecule expression. We found that the pri-miRNA format outperformed the other two formats. It produced mature miRNA at 2 orders of magnitude higher levels and silenced target genes with an efficiency comparable to that achieved with optimized miRNA mimic transfection.

RESULTS

Construction of Gene-Silencing Oncolytic Adenoviruses

To allow rapid introduction of gene-silencing molecules into the oncolytic adenovirus genome, we made use of Gateway recombination (Figure 1A). To this end, we constructed recipient plasmid pAdΔ24E3-DEST-R that carries the full-length genome of an AdΔ24-type oncolytic adenovirus with a Gateway Destination cassette inserted between the adenovirus E4 region and the right-hand inverted terminal repeat. This locale does not overlap with any adenovirus transcript. Expression cassettes for gene-silencing molecules can be introduced in pAdΔ24E3-DEST-R using a two-step cloning procedure. First, synthetic hairpin-encoding DNA fragments are inserted in entry plasmid pSHAG-1, which places transcription of the hairpin RNA under direction of the U6 promoter. Second, the hairpin expression cassette is transferred to the oncolytic adenovirus genome by *in vitro* recombination with pAdΔ24E3-DEST-R. Bacterial transformation of the recombination products and ampicillin selection yields only bacteria carrying plasmids with the hairpin-containing adenovirus. Upon plasmid isolation from these cells and PacI digestion, the released linear viral genome is transfected into human cancer cells to start virus propagation. Using this method, five recombinant viruses were made. In addition to an empty expression cassette control, made by recombining pSHAG-1 without insert with pAdΔ24E3-DEST-R, we made three viruses expressing a human microRNA-1 (miR-1) and one virus expressing a human microRNA-26b (miR-26b) precursor. Human miR-1 was chosen because this tumor-suppressor microRNA is expressed at very low levels in many cancer cells,[11] allowing sensitive detection of exogenous expression. miR-26b was used because of its assumed effect on adenovirus propagation in prostate cancer cells.[12] All inserted sequences are given in Table S1. miR-1 was expressed in three different formats, each predicted to be processed by the RNAi machinery into mature hsa-miR-1-3p (Figure 1B). To create shmiR-1 (i.e., a miR-1 precursor in shRNA format), the sequences in the stem of the firefly luciferase shRNA that we previously expressed from the genome of an AdΔ24-type virus[4] were replaced by those of the hsa-miR-1 mimic duplex used by Lim et al.[13] The sequences in the shmiR-1 stem are mostly complementary, with a mismatch near the 5′ end of the miR-1-3p sense strand to promote its incorporation into the RNA-induced silencing complex (RISC).[13] A miR-1 precursor in pre-miRNA-like format was derived from hsa-miR-1-2 to include the endogenous hsa-miR-1-2 loop and stem until the end of the mature hsa-miR-1-3p sequence, with its natural mismatches between the sense and antisense strands. The shmiR-1 and pre-miR-1 constructs have their 5′ start nucleotide set to be guanine (G indicated in bold in Figure 1B), to accommodate the transcription start preference of the U6 promoter.[14] At the 3′ end of the hairpin, following the cytosine complementary to the 5′ guanine, a track of 5 thymines was added to direct proper transcription termination leaving 2 or more uracils in the 3′ transcript overhang as a substrate for processing by Dicer.[15] The third miR-1 precursor, in pri-miRNA format, comprises the endogenous hsa-miR-1-1 sequences with approximately 120 nucleotides of flanking sequences on both sides of the hairpin. Also here, a guanine was placed at the +1 position after the U6 promoter and a 5-thymine tail was added. For this construct, the pri-miR-1-1 sequence was preferred over that of pri-miR-1-2, because the latter contains a stretch of 5 thymines in one of the flanks that would serve as a premature termination signal for RNA polymerase III. Finally, following the same design strategy, a pri-miR-26b encoding adenovirus was constructed. The predicted secondary folding structures (generated with RNAfold Webserver[16]) of the pri-miR-1 and pri-miR-26b transcripts are shown in Figures 1C and 1D, respectively.

Comparison of Three Different Formats for miRNA Expression from the Adenovirus Genome

To investigate which expression format is most suited for effective gene silencing in the context of oncolytic adenovirus replication, the newly constructed miR-1 precursor-carrying viruses were compared for their oncolytic replication potency and stable miRNA expression in human cancer cells. The three viruses exhibited similar titers as the empty expression cassette control virus when produced in A549 non-small cell lung cancer cells (Table S2). Their oncolytic potency was determined by infecting A549 cells with virus dilution series and measuring cytotoxicity 7 days post infection. A549 cells were chosen for these experiments because they efficiently support human adenovirus propagation, allowing sensitive detection of differences in oncolytic potency. The virus concentration required to kill 50% of the

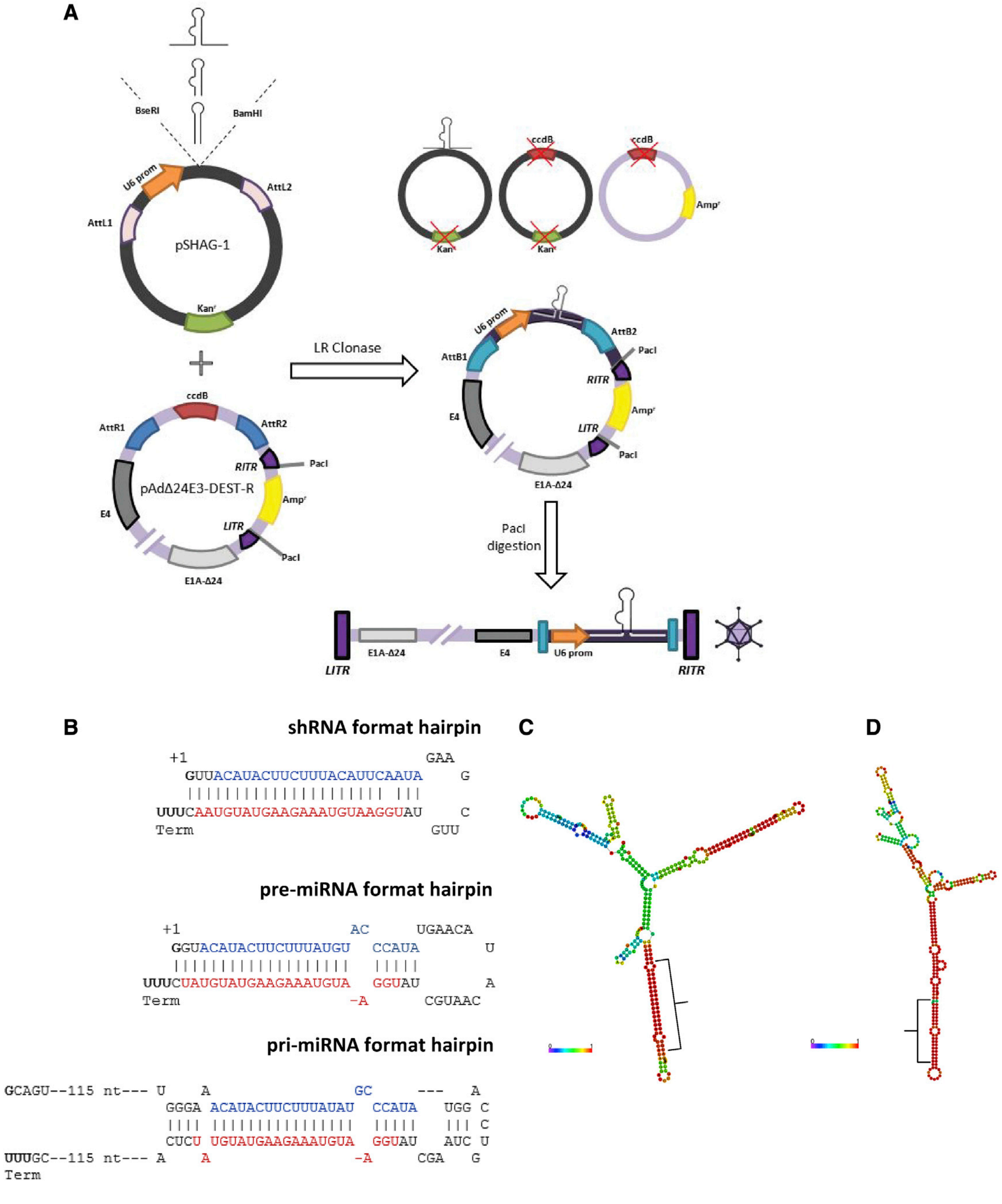

Figure 1. Construction of Gene-Silencing Oncolytic Adenoviruses

(A) Schematic strategy for rapid production of oncolytic adenoviruses with incorporated RNAi molecule expression cassette. (B) RNA transcript sequences of the three different miR-1 precursor designs. Mature and passenger strand sequences are shown in red and blue, respectively. (C and D) Predicted optimal secondary folding structures of adenovirus-encoded hsa-pri-miR-1 (C) and hsa-pri-miR-26b (D), with the microRNA stem-loops pointing down. Predicted structures are based on minimum free energy and base-pairing probability calculation using RNAfold WebServer.[16] The positions of the mature miRNA sequences are indicated.

cells (EC_{50}) values derived from dose-response curves (Figure 2A) were used to calculate average oncolytic potencies ($1/EC_{50}$), which were 186 for AdΔ24E3-U6 (95% probability range, 137–254), 25 for AdΔ24E3-U6.shmiR-1 (range, 18–34), 15 for AdΔ24E3-U6.pre-miR-1 (range, 7–35), and 46 for AdΔ24E3-U6.pri-miR-1 (range, 30–72). Thus, insertion of hairpin-encoding sequences decreased oncolytic virus cytotoxicity 4- to 12-fold. While the pri-miR-1-carrying virus exhibited the highest oncolytic potency of the three

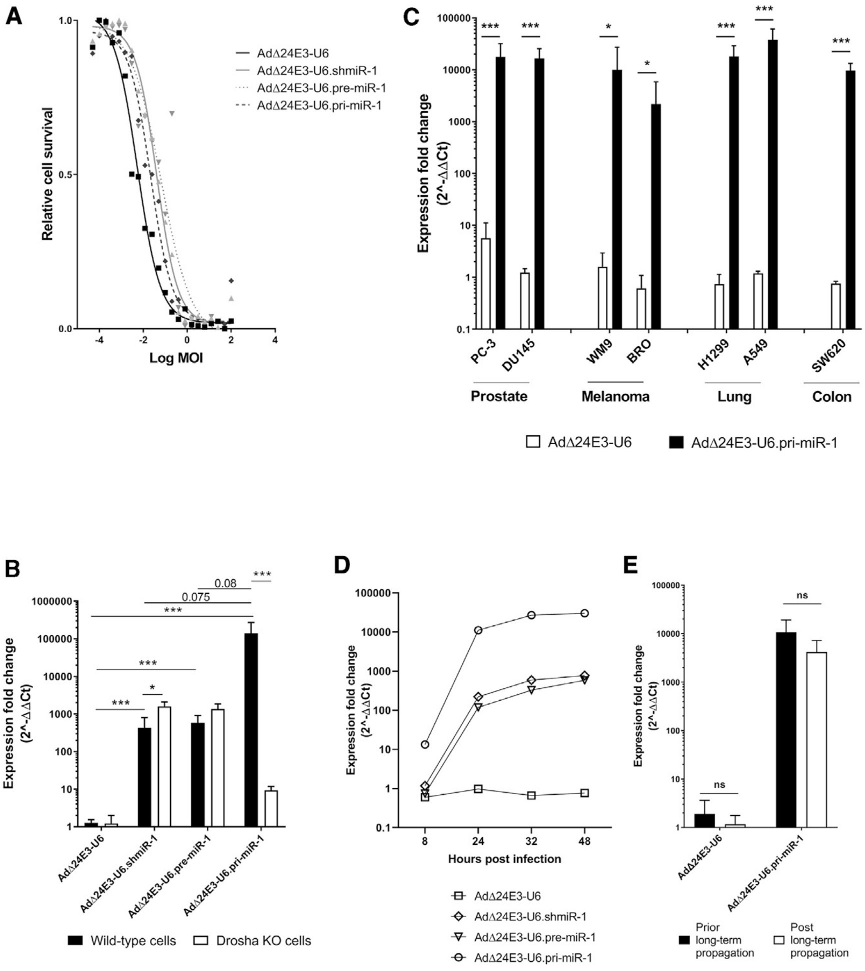

Figure 2. Analysis of Oncolytic Adenoviruses Expressing Different MicroRNA Precursor Molecules

(A) Dose-response cytotoxicity analysis of oncolytic adenoviruses expressing different miR-1 precursor molecules on A549 lung cancer cells. Data shown are means of two independent experiments in triplicate. (B) Quantitative RT-PCR analysis of mature miR-1-3p expression in HCT116 parental and *Drosha* knockout (KO) colorectal carcinoma cells infected with oncolytic adenoviruses expressing different miR-1 precursor molecules. (C) Expression of mature miR-1-3p in different cancer cell lines infected with AdΔ24E3-U6 or AdΔ24E3-U6.pri-miR-1. (D) Time course analysis of mature miR-1-3p expression in A549 cells infected with oncolytic adenoviruses expressing different miR-1 precursor molecules. (E) Comparison of mature miR-1-3p expression in HCT116 cells infected with AdΔ24E3-U6 or AdΔ24E3-U6.pri-miR-1, before and after 6 weeks of virus propagation. Data shown in (B)–(E) are means of three independent experiments with cells infected at 100 IU/cell in duplicate and are given relative to uninfected cells.

insert-containing viruses, differences were not significant, indicating that their hairpin sequence insertions were similarly compatible with adenovirus biology. This allows direct comparison of miRNA expression in infected cancer cells, without a potential confounding effect of temporal differences in virus-host interactions.

Next, we compared the viruses for their mature miR-1 expression in infected cells and studied the involvement of DROSHA in exogenous miR-1 processing. To this end, an isogenic pair of wild-type and *Drosha* knockout HCT116 colorectal cancer cell lines was infected with the different viruses, and 32 h later mature miR-1-3p was measured by qRT-PCR. The cells were infected at a high multiplicity of infection (MOI) of 100 IU/cell. At this MOI, cells are efficiently infected (Figure S2), which allows monitoring of miRNA expression during a single virus life cycle. MicroRNA expression was measured 32 h after infection, because we had previously seen that transgene expression from replicating adenovirus vectors by then had reached a plateau, and knockdown of firefly luciferase by adenovirus-encoded shRNA was evident.[4,17] As can be seen in Figure 2B, in wild-type HCT116 cells, all three hairpin formats were expressed from the adenovirus genome and processed into mature miR-1-3p, reaching levels that were substantially higher than the endogenous miR-1-3p levels measured in uninfected cells or in cells infected with empty control virus AdΔ24E3-U6 ($p < 0.001$). Strikingly, the virus carrying miR-1 in the pri-miRNA format clearly stood out, yielding on average more than two orders of magnitude higher levels of mature miR-1-3p than the other two formats. Although this difference did not reach significance ($p = 0.08$), it was highly reproducible (Figure S3). High levels of mature miR-1-3p expression were also measured when AdΔ24E3-U6.pri-miR-1 was used to infect other cancer cell lines representing several different tumor types (Figure 2C). To investigate the course of miR-1 expression after host cell infection, A549 cells were infected with the three miR-1-expressing viruses, and mature miR-1-3p was quantified at various time-points between 4 and 48 h post infection. As can be seen in Figure 2D, miR-1-3p expression from all three precursor formats rapidly increased during the first day after infection and reached maximum values after 32–48 h. As expected, miRNA expression levels in infected cells correlated with the MOI (Figure S4). *Drosha* knockout almost completely abolished mature miR-1-3p expression in AdΔ24E3-U6.pri-miR-1-infected cells ($p < 0.001$; Figures 2B and S3), confirming that processing of the pri-miRNA by DROSHA was essential for efficient entry into the host cell miRNA machinery. In contrast, processing of shmiR-1 and pre-miR-1 was not inhibited by *Drosha* knockout, which is in line with their DROSHA-independent design. In fact, *Drosha* knockout seemed to slightly elevate mature miR-1-3p production in AdΔ24E3-U6.shmiR-1- and AdΔ24E3-U6.pre-miR-1-infected cells, suggesting that *Drosha* knockout reduced competition by endogenous miRNAs for use of the RNAi machinery.

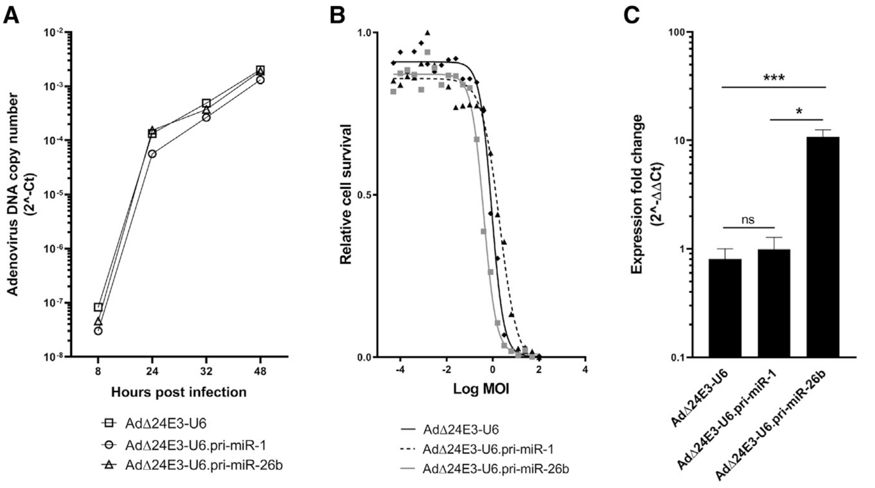

Figure 3. Replication of Oncolytic Adenoviruses Expressing miR-1 or miR-26b in Cancer Cells

(A) Analysis of adenovirus DNA copy numbers in A549 cells infected with AdΔ24E3-U6, AdΔ24E3-U6.pri-miR-1, or AdΔ24E3-U6.pri-miR-26b by qPCR for adenovirus packaging signal sequences. Data shown are the means of two independent experiments in duplicate. (B) Dose-response cytotoxicity analysis of AdΔ24E3-U6, AdΔ24E3-U6.pri-miR-1, and AdΔ24E3-U6.pri-miR-26b on A549 cells. Data shown are means of two independent experiments in triplicate. (C) Analysis of mature miR-26b-5p levels in A549 cells 32 h after infection with AdΔ24E3-U6, AdΔ24E3-U6.pri-miR-1, or AdΔ24E3-U6.pri-miR-26b. Data shown are means of three independent experiments in duplicate and are given relative to uninfected cells.

Having established the superiority of the pri-miRNA format, we further investigated whether miR-1 expression from AdΔ24E3-U6.pri-miR-1 remains stable upon long-term virus propagation. To this end, HCT116 cells were infected with AdΔ24E3-U6 and AdΔ24E3-U6.pri-miRNA-1 adenoviruses at a low MOI of 0.25 IU/cell and cultured until near-complete cytopathic effect (CPE) was observed. Viruses were then harvested, diluted, and used to infect newly seeded cells. This procedure was continued for a total of 6 weeks, equaling an estimated 15 virus life cycles. The virus progeny in the final harvest was titrated and used to measure mature miR-1-3p expression in HCT116 cells in a side-by-side comparison with the start material (Figure 2E). miR-1-3p expression did not change upon prolonged viral replication, showing that the functional silencing cassette was stably maintained in the adenovirus genome. The same observation was made when AdΔ24E3-U6.pri-miR-1 was propagated on A549 cells (not shown).

Expression of a Different pri-miRNA from the Oncolytic Adenovirus Genome

We also constructed AdΔ24E3-U6.pri-miR-26b, using the same cloning procedure. In contrast to miR-1, expression of miR-26b could perhaps influence virus replication. We previously observed that miR-26b overexpression in prostate cancer cell lines promoted adenovirus-induced cell death and increased adenovirus progeny release, resulting in larger plaque sizes.[12] While this had not been investigated in cells other than prostate cancer cells, similar effects could perhaps be anticipated in the cells used in this study. Interestingly, AdΔ24E3-U6.pri-miR-26b grew to 30- to 55-fold higher titers in A549 cells than the other four viruses used in this study (Table S2). In fact, the titer of the unpurified AdΔ24E3-U6.pri-miR-26b virus batch was higher than we usually obtain for any oncolytic adenovirus. To investigate possible mechanisms of miR-26b-mediated enhancement of adenovirus production, we measured adenovirus DNA replication and cytotoxic virus replication on A549 cells, in comparison to the empty control virus and the pri-miR-1-expressing virus. Figure 3A shows that adenovirus DNA copy numbers in cells infected with AdΔ24E3-U6, AdΔ24E3-U6.pri-miR-1, and AdΔ24E3-U6.pri-miR-26b increased with similar rates. In addition, the three viruses exhibited similar oncolytic potencies on A549 cells (Figure 3B). Thus, the considerable increase in infectious progeny virus production by miR-26b expression remains unexplained. Figure 3C shows that infection of A549 cells with AdΔ24E3-U6.pri-miR-26b increased the levels of mature miR-26b-5p 10-fold compared to controls. The apparently smaller increase of mature miRNA expression compared to that seen with AdΔ24E3-U6.pri-miR-1 (approximately 100,000-fold; Figure 2) is explained by much higher endogenous levels of miR-26b-5p than miR-1-3p (estimated 10,000-fold difference, data not shown).

Efficiency of Adenovirus-Encoded miRNA Processing by the RNAi Machinery and Silencing of Target Genes in Host Cells

We next compared the levels of mature miRNA delivered by pri-miRNA-expressing adenoviruses to those that are achieved by transfection with miRNA mimics. HCT116 cells were infected with AdΔ24E3-U6.pri-miR-1 or AdΔ24E3-U6.pri-miR-26b or were transfected with miR-1 or miR-26b mimics under optimized conditions. After 32 h, RNA was isolated and mature miRNA was quantified by qRT-PCR. As can be seen in Figures 4A and 4B, both viruses elevated the expression levels of their encoded miRNA, but these levels were considerably lower than was achieved by transfecting miRNA mimics. High-level exogenous pri-miRNA expression could potentially inhibit processing of endogenous miRNA in the host cell. However, exogenous overexpression of pri-miR-1 did not reduce endogenous miR-26b processing (Figure 4B), and, vice versa, exogenous overexpression of pri-miR-26b did not reduce endogenous miR-1 processing (Figure 4A). Because efficient miRNA mimic transfection or high pri-miRNA expression could perhaps exceed RISC-loading capacity, we next investigated binding of adenovirus-encoded miRNA-26b and miR-26b mimics to the Argonaut (AGO) RNA binding protein of the RISC. To this end, small RNA associated with AGO proteins was isolated using peptide-based pull-down[18] (Figure S5A). Total cellular RNA and pan-AGO-bound RNAs were analyzed by miR-26b-5p (mature strand)-specific and miR-26b-3p (passenger strand)-specific qRT-PCR, and amounts were compared (Figure S5B). All cells contained more mature miR-26b-5p than

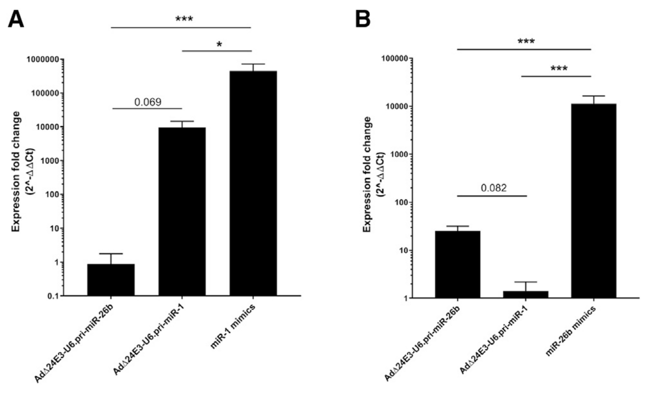

Figure 4. Processing of Introduced miRNA Precursor Molecules by the Host Cell RNAi Machinery

(A) Comparison of mature miR-1-3p levels in HCT116 cells treated with miR-1 mimics, pri-miR-1-expressing virus, or control pri-miR-26b-expressing virus. (B) Comparison of mature miR-26b-5p levels in A549 cells treated with miR-26b mimics, control pri-miR-1-expressing virus, or pri-miR-26b-expressing virus. Data shown are means of three independent experiments in duplicate and are given relative to uninfected cells.

passenger miR-26b-3p. Under most conditions, AGO pull-down slightly enriched for mature miR-26b-5p. Only upon miR-26b-mimic transfection, less (approximately 30%) miR-26b-5p mimic RNA was found associated with Argonaut proteins. Nevertheless, much more miR-26b-5p was loaded into RISC in miR-26b mimic-transfected cells than in AdΔ24E3-U6.pri-miR-26b-infected cells. Endogenous miR-26b, oncolytic adenovirus-encoded miR-26b hairpins, and transfected miR-26b mimic duplexes exhibited a strong preference for incorporation of the mature miR-26b-5p strand into RISC. Exogenous expression of oncolytic adenovirus-encoded pri-miR-1 did not detectably influence RISC loading of endogenous miR-26b. Thus, RISC was properly loaded with the mature antisense strand of adenovirus-expressed pri-miRNA, and the RISC-loading capacity exceeded the amount of adenovirus-produced microRNA.

To investigate if the amounts of adenovirus-encoded miRNA expressed and processed in the RNAi machinery were sufficient for effective gene silencing, we measured expression levels of validated miR-1 and miR-26b target genes in infected cancer cells.[19–22] MDA-MB-231 breast cancer cells, which express high endogenous levels of miR-1 target genes FOXP1 and MET as well as miR-26b target gene PTGS2, were infected with AdΔ24E3-U6.pri-miR-1 or AdΔ24E3-U6.pri-miR-26b, and FOXP1, MET, and PTGS2 mRNA was quantified by qRT-PCR. In these experiments, the two viruses served as mutually irrelevant controls. Figure 5A shows that miR-1-3p and miR-26b-5p were expressed in MDA-MB-231 cells infected with the pri-miRNA-expressing viruses. Expression increased exponentially over a 2-day period, leveling off at 48 h post infection. Both viruses exhibited the anticipated target gene knockdown, with AdΔ24E3-U6.pri-miR-1 infection decreasing FOXP1 and MET expression and AdΔ24E3-U6.pri-miR-26b infection decreasing PTGS2 expression (Figure 5B). Although there was considerable inter-experimental variation, which was in line with the observed variation in miRNA expression levels, specific knockdown was significant (all: $p < 0.01$). To investigate if oncolytic adenovirus-encoded exogenous pri-miRNA or VA-RNA expression could saturate and thereby inhibit the miRNA biogenesis pathway, cells were infected with AdΔ24E3-U6 or AdΔ24E3-U6.pri-miR-1 and non-target PTGS2 mRNAs was quantified or with AdΔ24E3-U6 or AdΔ24E3-U6.pri-miR-26b and non-target FOXP1 and MET mRNAs were quantified. As can be seen in Figure 5C, PTGS2 and MET expression appeared similarly, but not significantly, increased upon infection with both viruses. Thus, there was a trend suggesting collateral inhibition by VA-RNA expression but not by exogenous pri-miRNA expression. To confirm that the observed gene silencing was caused by direct miRNA-mediated knockdown, we used an HCT116 reporter cell line expressing an optimized *Renilla* luciferase variant (RenSP) with human FOXP1-3′UTR. These cells were infected with the three miR-1 precursor-expressing oncolytic adenoviruses or transfected with miR-1 mimics, and knockdown was measured after 24 and 32 h (Figure 5D). As can be seen, all treatments significantly reduced luminescence intensity. Notably, AdΔ24E3-U6.pri-miR-1 induced the most efficient gene silencing. At 32 h post infection, it even exceeded that of miR-1 mimic transfection. This was remarkable in view of the much higher mature miR-1-3p levels observed upon mimic transfection (Figure 4A). In addition, in AdΔ24E3-U6.pri-miR-1-infected cells, target gene silencing became apparent sooner after infection than in cells infected with the other two viruses. At 24 h after treatment, luminescence was decreased upon infection with AdΔ24E3-U6.pri-miR-1 or transfection with miR-1 mimics but not yet upon infection with the other two viruses. To discriminate between adenovirus and miR-1-dependent effects on RenSP-FOXP1-3′UTR expression, we compared luminescence in reporter cells infected with AdΔ24E3-U6.pri-miR-1 or AdΔ24E3-U6.pri-miR-26b (Figure 5E). AdΔ24E3-U6.pri-miR-26b infection did not significantly reduce luminescence. In contrast, AdΔ24E3-U6.pri-miR-1 infection silenced RenSP-FOXP1-3′UTR expression with similar efficiency as miR-1 mimic transfection ($p < 0.05$). Hence, the expression of adenovirus-encoded pri-miRNA and its processing in the RNAi machinery was sufficient to bring about gene silencing, with an efficiency and a velocity equal to or exceeding that of miRNA mimic transfection under optimized conditions.

DISCUSSION

Expression of RNAi silencing molecules from the genome of replicating viruses is considered a useful strategy to achieve more effective oncolytic virotherapy of cancer.[5,23] Useful microRNAs and target genes for this purpose are identified by functional genetic screening[24–27] or are chosen on the basis of their known functions.[6,7,23,28,29] While the tested approaches were generally successful, reported gene silencing efficiencies were variable. We envisioned that, depending on the target gene, more consistent knockdown could be required to achieve a therapeutic effect.

Although shRNA-like stem-loop vectors are most commonly used, RNAi molecules can be expressed in cells in different precursor

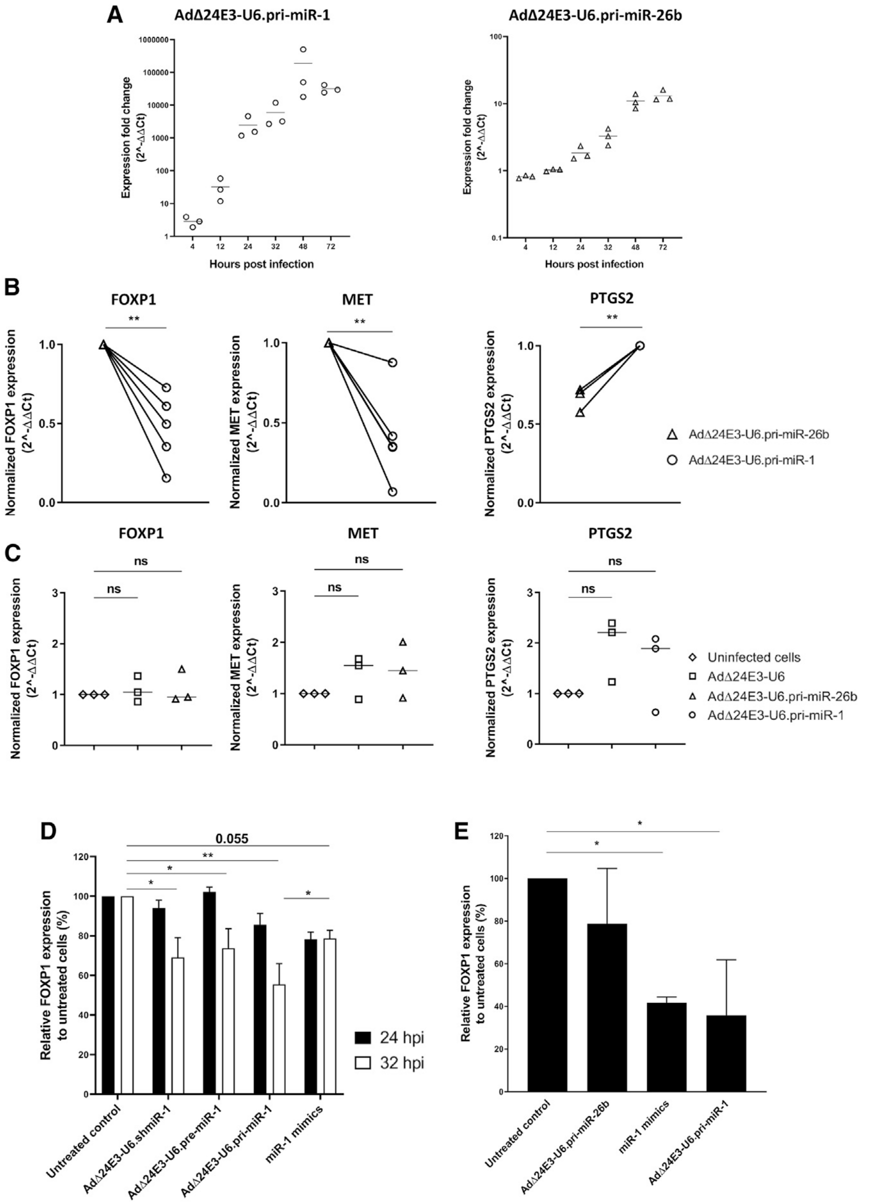

Figure 5. Silencing of Validated Target Genes by Adenovirus-Encoded miRNAs

(A) Time course of mature miRNA expression in MDA-MB-231 cells infected with AdΔ24E3-U6.pri-miR-1 or AdΔ24E3-U6.pri-miR-26b. Data shown are the individual results and means of three independent experiments, each measured in duplicate, and are given relative to uninfected cells. (B) Analysis of mRNA knockdown of miR-1 targets FOXP1 and MET and of miR-26b target PTGS2 in MDA-MB-231 cells infected with pri-miRNA-expressing adenoviruses. Data are from 5 or 3 independent experiments, each measured in duplicate, and are normalized to the expression measured in irrelevant virus-infected cells. Paired t test was used to test for significance of mRNA knockdown. (C) Comparison of collateral inhibition of gene silencing in cells infected with miRNA-expressing adenovirus. MDA-MB-231 cells were infected with AdΔ24E3-U6, AdΔ24E3-U6.pri-miR-1, or AdΔ24E3-U6.pri-miR-26b, and expression of non-target genes was quantified. Data are from 3 independent experiments, each measured in duplicate, and are normalized to the expression measured in uninfected cells. (D) Analysis of miRNA-mediated silencing of FOXP1 by measuring luminescence in an HCT116 reporter cell line carrying a FOXP1-3′UTR-RenSP plasmid, at 24 and 32 h after infection with miR-1 precursor-expressing adenoviruses or transfection with miR-1 mimics. Data shown are means + SD from three independent experiments done in triplicate. Repeated-measures one-way ANOVA was used to test for differences between groups. The results shown are from the analysis done on the data at 32 h after treatment. (E) Analysis as in (D), measuring luminescence 24 h after infection with the pri-miR-1 or pri-miR-26b-expressing adenovirus or transfection with miR-1 mimics. Data shown are means + SD from a representative experiment done in triplicate.

formats. Recently, three different formats were compared in the context of replication-defective adenovirus shuttle plasmids.[30] Expression in pre-miRNA or pri-miRNA format or as two single-strand complementary RNAs yielded similar expression levels and knockdown efficiencies. As far as we know, however, a side-by-side comparison of different silencing formats has not been made using replication-competent adenoviruses. In this context, virus-host interactions are expected to impact miRNA biogenesis, in particular via VA-RNA expression,[10] and therefore the processing not only of endogenous miRNAs but also of exogenous virus-encoded silencing molecules could be inhibited (Figure S1). In addition, because oncolytic adenoviruses in contrast to replication-defective adenovirus vectors kill their host cancer cell, effective gene knockdown should be achieved rapidly after infection to be meaningful. Therefore, we set out to compare different precursor formats expressed from the genome of oncolytic adenoviruses during their replication in cancer cells. In contrast to what was previously reported for transfected replication-defective adenovirus shuttle plasmids,[30] we found that expression from a conditionally replicating adenovirus genome in pri-miRNA format yielded 2 orders of magnitude higher production of mature silencing molecules than expression in shRNA or pre-miRNA format and that this increased production resulted in the most efficient and rapid knockdown of endogenous target genes. A possible explanation for the striking difference between the observation with replication-defective adenovirus vector plasmids made before[30] and with replicating adenoviruses made here is competition between VA-RNA and miRNA precursors for processing in the miRNA biogenesis pathway (Figure S1). pri-miRNAs enter the canonical miRNA biogenesis pathway in the nucleus by binding to the microprocessor complex consisting of multiple nuclear proteins, including DROSHA.[31] The DROSHA-cleaved intermediate

pre-miRNA products are subsequently handed over to the nuclear export receptor exportin 5 (XPO5) for further processing by the cytoplasmic RNAi machinery. It has been proposed that interleukin enhancer-binding factor 3 (ILF3) shuttles pre-miRNAs from the microprocessor complex to XPO5.[32] In contrast, VA-RNAs, shRNAs, and pre-miRNAs interact with XPO5 for nuclear export without the aid of the microprocessor complex and ILF3. It is tempting to speculate that the ILF3-mediated delivery of pri-miRNAs to XPO5 is more effective, imparting pri-miRNAs, but not shRNAs and pre-miRNAs, a competitive advantage over VA-RNAs to enter the miRNA biogenesis pathway.

Competition of endogenous pri-miRNAs with adenovirus-encoded miRNAs was probably very small. This can be deduced from the observation that expression of shRNA or pre-miRNA precursors, which do not require DROSHA for processing, resulted in only slightly more mature miRNA produced in *Drosha*-knockout cells than in *Drosha*-wild-type cells. Only the latter cells process endogenous pri-miRNAs via the canonical pathway that could perhaps compete with the adenovirus-encoded molecules. Endogenous pri-miRNAs thus did not saturate XPO5 or the cytoplasmic RNAi machinery, leaving sufficient processing capacity for adenovirus-encoded precursors. In addition, miRNA mimic transfection resulted in much more RISC loading than could be reached with adenovirus-pri-miRNA infection. Hence, also at the final effector level, the capacity of the miRNA biogenesis pathway was not limiting exogenous pri-miRNA processing. Conversely, a possible concern of high-level expression of exogenous miRNA precursors is oversaturation of the miRNA biogenesis pathway, reducing the processing of endogenous miRNAs (Figure S1). While this possible collateral effect of oncolytic adenovirus-encoded pri-miRNA expression was not investigated extensively, our results suggest that inhibition of endogenous miRNA biogenesis does not occur prominently. Exogenous pri-miRNA expression did not detectably reduce endogenous miRNA processing. It also did not increase expression of a non-target miRNA-regulated gene. Thus, in the context of adenovirus replication and VA-RNA expression, the potential inhibitory effect on the RNAi machinery by additionally expressing an exogenous pri-miRNA is probably limited.

Although adenovirus-encoded pri-miRNA expression clearly stood out compared to pre-miRNA or shRNA expression, the amount of mature miRNA produced in adenovirus-pri-miRNA-infected cells and loaded into RISC was considerably lower than could be reached by miRNA mimic transfection. This suggests that there is still room for further improvement. For endogenous miRNAs, it has been found that the efficiency of pri-miRNA cleavage in the microprocessor complex rather than their primary transcription is a key regulatory step in the biogenesis.[33] Although it is not known if this is also true for adenovirus-encoded pri-miRNAs, efforts to improve miRNA production are therefore best focused on hairpin sequence modifications that increase processing efficiency rather than primary transcription efficiency. However, despite the large difference in mature miRNA abundance in pri-miRNA-expressing adenovirus-infected cells and miRNA mimic-transfected cells, gene-silencing efficiency measured in the former cells was at least equivalent. This suggests that attempts to further increase miRNA production from the adenovirus genome might not be urgent.

Expression of mature miRNA from adenovirus-encoded pri-miRNA was highly dependent on DROSHA, showing that processing mainly occurred via the canonical miRNA biogenesis pathway. Nevertheless, a small fraction above background was detected in *Drosha*-knockout cells, suggesting that non-canonical DROSHA-independent miRNA biogenesis of adenovirus-encoded pri-miRNA could also occur. Several DROSHA-independent miRNA biogenesis pathways have been identified that could perhaps explain this observation.[31] The abundance of non-canonically processed pri-miRNAs was, however, so low that their contribution to gene silencing can probably be neglected.

In our experiments, we also sought to develop a simple construction method for gene-silencing oncolytic adenoviruses, where different silencing cassettes could be inserted into the relatively large adenovirus genome. For this, we introduced a Gateway recombination destination cassette into the full-length genome of an oncolytic adenovirus with E1A-Δ24 modification.[34] We tested the utility of this cloning system by generating five different viruses expressing—in addition to a negative control—two different human microRNAs in different precursor formats. All viruses could easily be generated and propagated, suggesting that the cloning system will be of use to construct new oncolytic viruses silencing target genes identified in functional genetic screens. A similar approach, based on recombination in bacteria rather than *in vitro* as we did here, was recently used to generate a pooled miRNA expression library in replication-competent adenoviruses.[26] The simplicity of these cloning methods thus allows production of not only specific gene-silencing viruses but also gene-silencing libraries. Notably, we found that regardless of the precursor format used, introduction of short hairpin-encoding sequences had a small negative impact on the replication properties of the virus. Introduction of the most effective silencing molecules in pri-miRNA format reduced oncolytic replication potency on average 4-fold. Although the effect was small, it was significant and can therefore not be neglected. The benefit of expressing therapeutic miRNAs from the virus genome, at least using the designs tested by us, should thus be balanced against the modestly reduced cancer cell-killing rate of the virus. Modifications to the adenovirus genome that inhibit replication could impose a negative selection pressure, causing outgrowth of revertants lacking the modification. At least in the extent of our *in vitro* experiments, this did not appear to take place, as miRNA expression remained stable during virus propagation.

Our experiments were not designed to investigate effects of expressed miRNAs on adenovirus biology. Nevertheless, we found that the oncolytic adenovirus expressing miR-26b produced much higher titers than empty control or miR-1-expressing adenoviruses on A549 cells. Although this observation could not be explained by differences in the rate of virus replication, it complements our previous observation that

miR-26b overexpression in prostate cancer cells enhanced propagation of wild-type human adenovirus serotype 5.[12] The latter observation was made on cells that already overexpressed miR-26b before they were infected with adenovirus. Thus, in those experiments miR-26b could influence the entire adenovirus life cycle. In our current work, miR-26b was expressed from the virus genome and target gene knockdown was delayed until the late phase of adenovirus infection. More research is needed to assess the impact of miR-26b on adenovirus biology, with particular attention for the timing of miRNA expression during the virus life cycle.

In conclusion, we developed a platform for easy construction of oncolytic adenoviruses that express high levels of RNAi molecules of choice. These viruses induce effective gene knockdown in infected cancer cells. The platform offers opportunities to design next-generation viruses for more effective oncolytic virotherapy of cancer.

MATERIALS AND METHODS

Cell Lines

A549 non-small cell lung cancer cells, MDA-MB-231 breast cancer cells, and 911 adenovirus E1-complementing cells were grown in Dulbecco's modified Eagle's medium (DMEM) (Sigma-Aldrich, St. Louis, MO) supplemented with 10% fetal bovine serum (FBS) (GIBCO-Thermo Fisher Scientific, Waltham, MA) and antibiotics (100 IU/mL penicillin and 100 μg/mL streptomycin) (Sigma-Aldrich). HCT116 colorectal cancer cells—a parental and *Drosha* knockout clone[35] (product numbers BP1230983 and BP1230984, respectively)—were obtained from the Korean Collection for Type Cultures (KCTC, Jeollabuk-do, South Korea) and grown in McCoy's 5A medium (Lonza, Basel, Switzerland), supplemented with 10% FBS, antibiotics, and 2 mM glutamine. H1299 non-small cell lung cancer cells, SW620 colorectal cancer cells, PC-3 and DU145 prostate cancer cells, and Mel-BRO and WM9 melanoma cells were grown in RPMI 1640 medium (Sigma-Aldrich) supplemented with 10% FBS and antibiotics. All cells were maintained at 37°C in 5% CO_2. FOXP1-3′UTR reporter cells were made by co-transfection of LightSwitch FOXP1-3′UTR GoClone reporter plasmid (Switchgear Genomics, Menlo Park, CA) and pTK-Hyg Hygromycin selection plasmid (Clontech, Mountain View, CA) into HCT116 cells using FuGene HD Transfection Reagent (Promega, Madison, WI) according to the manufacturer's instructions, followed by selection of individual transfected clones in complete McCoy's 5A medium (supplemented as mentioned above) with additional 75 μg/mL Hygromycin B (Carl Roth, Karlsruhe, Germany). Individual resistant clones were maintained in the same medium, and expression of *Renilla* luciferase from the GoClone reporter plasmid was confirmed using the *Renilla* Luciferase Assay System (Promega).

Construction of Recombinant Adenovirus Genomes with miRNA Expression Cassettes

First, microRNA-encoding sequences were inserted through ligation into BseRI/BamHI-digested Gateway entry clone pSHAG-1 (generously provided by Dr. G.J. Hannon, Cold Spring Harbor Laboratory, NY; http://hannonlab.cshl.edu/plasmids/pSHAG_maps.gif). pSHAG-1 contains a U6 promoter-driven expression cassette flanked by the Gateway *att*L1 and *att*L2 recombination sites, such that the expression cassette can be transported into destination plasmid vectors using the Gateway system (Invitrogen, Carlsbad, CA). Synthetic DNA encoding miRNA in short hairpin miRNA mimic or pre-miRNA format was purchased from Dharmacon Thermo Scientific as complementary single-strand oligonucleotides, which were annealed to create duplexes with BseRI and BamHI compatible overhangs and then ligated into pSHAG-1. Plasmids carrying synthetic DNA sequences encoding pri-miRNAs encompassing the stem-loop with at least 119 nucleotides of flanking sequences on both sides (based on the human Genome Reference Consortium build 37 [GRCh37]) and flanking BseRI and BamHI restriction sites for pri-miR-1 or BsrDI and BamHI restriction sites for miR-26b were purchased from Thermo Fisher Scientific GeneArt (Regensburg, Germany). After digestion with BseRI or BsrDI (creating a 5′-CG-overhang compatible with BseR1-digested pSHAG-1) and BamHI, the released insert was ligated into BseRI/BamHI-digested pSHAG-1. All synthetic insert sequences (hsa-miR-1 mimic, hsa-pre-miR-1-2, hsa-pri-miR-1-1, and hsa-pri-miR-26b) including a 3′ TTTTT tail used herein are listed in Table S1.

A recipient vector carrying a Gateway recombination destination cassette between the adenovirus E4 region and the right-hand ITR was made to allow transfer of miRNA expression cassettes to the adenoviral genome. To this end, the construct pEndK/SpeI[4] was used. pEndK/SpeI carries Ad5 map units 0–7 and 93–100 separated by a unique KpnI site, PacI restriction sites flanking the two Ad5 ITRs, and a unique SpeI site that was introduced by changing Ad5 nucleotide 35813 from A to T by site-directed mutagenesis. pEndK/SpeI was made compatible with the Gateway system by ligating the Gateway destination cassette rfa (Gateway Vector Conversion System; Life Technologies Invitrogen) as a blunt fragment into the SpeI site (filled in with Klenow polymerase). A plasmid was selected that contained the Gateway destination cassette with the coding sequence of the ccdB gene on the adenovirus R strand and was designated pEndK/DEST-R. Next, Ad5-Δ24E3[34] linear dsDNA was isolated from virions and recombined with linearized pEndK/SpeI in *E. coli* BJ5183 bacteria to obtain plasmid clone pAdΔ24E3, from which full-length AdΔ24E3 DNA was released by PacI digestion. This DNA was recombined in *E. coli* BJ5183 bacteria with KpnI-digested pEndK/DEST-R to obtain pAdΔ24E3-DEST-R. pAdΔ24E3-DEST-R is propagated in *E. coli* strain STBL2-DB3.1, which contains a gyrase mutation that renders it resistant to the lethal effects of the CcdB protein allowing propagation of plasmids carrying the ccdB gene in the DEST cassette.

Empty and miRNA-encoding U6-driven expression cassettes were transferred from pSHAG-1 to pAdΔ24E3-DEST-R via an *in vitro* recombination reaction using the GATEWAY LR Clonase enzyme mix (Invitrogen) according to manufacturer's protocol.

Oncolytic Adenovirus Production and Characterization

Full-length adenovirus genomes with or without miRNA expression cassette were released via PacI digestion and transfected into A549

cells using Lipofectamine Plus (Invitrogen) transfection according to the manufacturer's instructions. Viruses were further propagated on A549 cells. Lysates were prepared when CPE was observed by freeze-thawing three times followed by clarification using centrifugation. Supernatants were stored at −80°C until use in experiments. Functional virus titers were determined by limiting dilution infection of 911 cells and hexon staining using the Adeno-X Rapid Titer Kit (Clontech). Titers of the produced virus batches are given in Table S2. Oncolytic potency was assessed by *in vitro* cytotoxicity assay. To this end, A549 cells were infected with two-fold serial virus dilutions, and 7 days later adherent cells were stained using the BCA Protein Assay kit (Thermo Fisher Scientific) as described before.[36] EC_{50} was calculated from dose-response curves by standard non-linear regression using a sigmoidal dose-response equation (GraphPad Prism, San Diego, CA).

Mature miRNA Expression Analysis Using qRT-PCR

RNA was isolated using TRIzol (Invitrogen) at the indicated time after infection with oncolytic adenovirus at MOI 100 or 32 h after transfection with 25 nM hsa-miR-1 mimic (Dharmacon, Lafayette, CA; cat. No. C-300585-05-0002) or hsa-miR-26b mimic (Dharmacon; cat. No. C-300501-07) using DharmaFect 2 (Dharmacon) according to the manufacturer's instructions. For preservation of miRNAs, 0.4 μg/mL glycogen (Invitrogen) was added to the aqueous phase during isolation. To quantify mature miR-1-3p expression, cDNA was prepared using the TaqMan MicroRNA Reverse Transcription kit (Applied Biosystems, Foster City, CA) and custom-ordered TaqMan MicroRNA Assays specific for human miR-1-3p or RNU48 (Applied Biosystems). The qPCR was performed on an Applied Biosystems 7500 Real-Time PCR system using TaqMan Universal Master Mix II, no UNG (Applied Biosystems), and custom-ordered TaqMan MicroRNA Assays as above. Mature miR-1-3p expression was normalized by RNU48 expression using the ΔCt method, and fold change over endogenous expression was calculated using the ΔΔCt method. To quantify mature miR-26b-5p or passenger strand miR-26b-3p expression, cDNA was prepared using the TaqMan MicroRNA Reverse Transcription kit and a custom miR-26b-5p-specific stem-loop (SL) primer[12] or miR-26b-3p-specific SL primer. The qPCR was performed on a Roche LightCycler 480 system using 5× HOT FIREPol EvaGreen qPCR Mix Plus (no ROX) (Solis BioDyne, Tartu, Estonia) using a custom miR-26b-5p-specific or miR-26b-3p-specific forward primer and a custom reverse primer directed against a sequence in the SL primer. Amplification of U6 small nuclear RNA (snRNA) was done for normalization as described.[12] Custom primers were purchased from Invitrogen and are listed in Table S3. Expression fold change was calculated as above. All experiments were performed in three independent runs, each including technical duplicates.

Long-Term Viral Propagation Assay

To investigate the stability of miRNA expression during adenovirus propagation, 3×10^5 HCT116 cells were seeded per well in a 6-well plate and infected with AdΔ24E3-U6 or AdΔ24E3-U6.pri-miR-1 at MOI 0.25. Viruses were allowed to propagate for up to 7 days, until 80%–90% of cells were in CPE. Viruses were harvested by collecting the cells and subjecting them to three freeze-thaw cycles. Harvested viruses were diluted 10,000 times and used to infect freshly seeded cells as above. This was repeated for a total of 6 cycles, after which a final amplification was done in a T75 flask. Propagated viruses were titrated as above, and titers are given in Table S2. HCT116 cells were infected at MOI 10 side by side with original stock virus and long-term propagated virus, and mature miR-1-3p expression was measured by qRT-PCR as above.

Analysis of Adenovirus DNA Replication Using qPCR

DNA was isolated from A549 cells at the indicated time after infection with oncolytic adenovirus at MOI 100 using the QIAamp DNA Blood Mini kit (QIAGEN). qPCR was performed on a Roche LightCycler 480 system using 5× HOT FIREPol EvaGreen qPCR Mix Plus (no ROX) and custom primers for the adenovirus packaging domain purchased from Invitrogen (Table S3). Reactions were done in duplicate and copy numbers are presented as 2^(-Ct) values.

Analysis of Target Gene Silencing Using qRT-PCR

MDA-MB-231 cells were infected with oncolytic adenoviruses at MOI-100, and RNA was isolated 32 h post infection as above. cDNA was prepared using the Cloned AMV First-Strand Synthesis Kit (Invitrogen) or FireScript RT cDNA Synthesis Kit (Solis BioDyne) according to the manufacturer's recommendations. qRT-PCR was performed on a Roche LightCycler 480 system using 5× HOT FIREPol EvaGreen qPCR Mix Plus (no ROX). Human *FOXP1*, *MET*, and *PTGS2* primers were purchased as custom oligonucleotides from Invitrogen (Table S3). Hs_GAPDH_2_SG QuantiTect Primer Assay (QIAGEN, Hilden, Germany) was used for *GAPDH*. All reactions were done in duplicate. *GAPDH*-normalized *FOXP1*, *MET*, or *PTGS2* gene expression was calculated using the ΔCt method; knockdown compared to irrelevant control virus-infected cells was calculated using the ΔΔCt method.

Analysis of Direct Silencing of FOXP1 Using a Reporter Assay

HCT116-FOXP1-3′UTR GoClone reporter cells were infected with oncolytic adenoviruses at MOI-100 as above or transfected with 25 nM hsa-miR-1-3p mimic as above. *Renilla* luciferase expression was measured after 24 and 32 h using the *Renilla* Luciferase Assay System (Promega) according to the manufacturer's instructions. Data are normalized by the luminescence measured in untreated reporter cells.

RISC Pull-Down Assay

We used a method similar to the one described by Hauptmann et al.[18] In short, GST-TNRC6B peptide was produced from plasmid pEC-K-3C-GST_TNRC6B_599-683, obtained from Gunther Meister (Regensburg, Germany), in *E. coli* BL21 (New England Biolabs, Ipswich, MA) and purified on a GST HiTrap 5 mL Column (GE Healthcare, Chicago, IL). Next, glutathione Sepharose 4B-conjugated beads (GE Healthcare) were pre-bound with the GST-TNRC6B peptide O/N at 4°C under rotation at a ratio of 200 μg/100 μL bead slurry in a final reaction volume of 1 mL of PBS. Unbound peptides were removed by washing with cold PBS twice. Cell lysates were prepared in NET buffer

(50 mM Tris-HCl pH 7.5, 150 mM NaCl, 5 mM EDTA, 0.5% NP-40, 10% glycerol, 1mM NaF, and 0.5 mM DTT) supplemented with protease inhibitor cocktail (Roche, Basel, Switzerland). Lysates were precleared at 10,000 × *g* for 10 min at 4°C, and the supernatant's protein concentrations were determined by a BCA assay in duplicate. Three milligrams of total protein was used as input for each reaction; these were mixed with 100 μL of pre-bound beads and incubated for 2 h at 4°C. Beads were subsequently washed 4 times in lysis buffer and 1 time in PBS. After washing, RNA was isolated from 70% of the material using TRIzol for 10 min on ice, and protein was isolated from 30% of the material by incubating at 70°C for 10 min in 2× LDS buffer (Thermo Fisher) for Western analysis, which was done as follows. LDS-containing isolates were separated on a 10% polyacrylamide gel and wet-blotted using a Tris-glycine-based buffer (25 mM Tris, 192 mM glycine, 0.05% SDS, 20% MeOH, pH 8.3). For immunodetection 1:1,000 rabbit-anti-Ago2 (C34C6, Cell Signaling Technology, Danvers, MA) and 1:2,000 goat-anti-rabbit immunoglobulins/horseradish peroxidase (Dako, Jena, Germany) antibodies were used. To visualize the blots, SuperSignal West Pico PLUS Chemiluminescent substrate (Thermo Scientific) was used.

Statistical Analyses

All statistical analyses were performed using GraphPad Prism (v 8.0.2) or R Studio version 3.6.2.[37] If datasets showed a skewed distribution, they were log-transformed to make the data distribution closer to the normal. Two-way ANOVA followed by Tukey's multiple comparisons test was used, unless specified otherwise. Data are expressed as mean + SD, and test results are summarized as "ns" for not significant, *p < 0.05, **p < 0.01, and ***p < 0.001.

SUPPLEMENTAL INFORMATION

Supplemental Information can be found online at https://doi.org/10.1016/j.omto.2020.10.012.

ACKNOWLEDGMENTS

The authors thank Lidija Milojković for technical assistance. This project obtained funding from the European Union's Horizon 2020 Research and Innovation Program with grant agreement number 643130.

AUTHOR CONTRIBUTIONS

Conceptualization, T.B., J.-W.v.G., and V.W.v.B.; Methodology, T.B., J.-W.v.G., M.J.L., D.K.-L., D.M.P., and V.W.v.B.; Formal Analysis, R.X.d.M.; Investigation, T.B., J.-W.v.G., and M.J.L.; Writing – Original Draft, T.B. and V.W.v.B.; Writing – Review & Editing, J.-W.v.G., M.J.L., R.X.d.M., D.K.-L., D.M.P., W.D., T.D.d.G.; Funding Acquisition, W.D., T.D.d.G., and V.W.v.B.; Supervision, W.D., T.D.d.G., and V.W.v.B. All authors read and approved the final manuscript.

DECLARATION OF INTERESTS

Tereza Brachtlova and Wenliang Dong are employees of ORCA Therapeutics B.V. Victor van Beusechem serves as CSO of ORCA Therapeutics B.V.

REFERENCES

1. de Gruijl, T.D., Janssen, A.B., and van Beusechem, V.W. (2015). Arming oncolytic viruses to leverage antitumor immunity. Expert Opin. Biol. Ther. *15*, 959–971.
2. Seymour, L.W., and Fisher, K.D. (2016). Oncolytic viruses: finally delivering. Br. J. Cancer *114*, 357–361.
3. Eager, R.M., and Nemunaitis, J. (2011). Clinical development directions in oncolytic viral therapy. Cancer Gene Ther. *18*, 305–317.
4. Carette, J.E., Overmeer, R.M., Schagen, F.H.E., Alemany, R., Barski, O.A., Gerritsen, W.R., and Van Beusechem, V.W. (2004). Conditionally replicating adenoviruses expressing short hairpin RNAs silence the expression of a target gene in cancer cells. Cancer Res. *64*, 2663–2667.
5. Brachtlova, T., and van Beusechem, V.W. (2018). Unleashing the Full Potential of Oncolytic Adenoviruses against Cancer by Applying RNA Interference: The Force Awakens. Cells *7*, 228.
6. Yoo, J.Y., Kim, J.-H., Kwon, Y.-G., Kim, E.-C., Kim, N.K., Choi, H.J., and Yun, C.O. (2007). VEGF-specific short hairpin RNA-expressing oncolytic adenovirus elicits potent inhibition of angiogenesis and tumor growth. Mol. Ther. *15*, 295–302.
7. Chu, L., Gu, J., Sun, L., Qian, Q., Qian, C., and Liu, X. (2008). Oncolytic adenovirus-mediated shRNA against Apollon inhibits tumor cell growth and enhances antitumor effect of 5-fluorouracil. Gene Ther. *15*, 484–494.
8. Hannon, G.J. (2002). RNA interference. Nature *418*, 244–251.
9. Pei, D.S., Di, J.H., Chen, F.F., and Zheng, J.N. (2010). Oncolytic-adenovirus-expressed RNA interference for cancer therapy. Expert Opin. Biol. Ther. *10*, 1331–1341.
10. Carnero, E., Sutherland, J.D., and Fortes, P. (2011). Adenovirus and miRNAs. Biochim. Biophys. Acta *1809*, 660–667.
11. Liu, C., Zhang, S., Wang, Q., and Zhang, X. (2017). Tumor suppressor miR-1 inhibits tumor growth and metastasis by simultaneously targeting multiple genes. Oncotarget *8*, 42043–42060.
12. Hodzic, J., Sie, D., Vermeulen, A., and van Beusechem, V.W. (2017). Functional screening identifies human miRNAs that modulate adenovirus propagation in prostate cancer cells. Hum. Gene Ther. *28*, 766–780.
13. Lim, L.P., Lau, N.C., Garrett-Engele, P., Grimson, A., Schelter, J.M., Castle, J., Bartel, D.P., Linsley, P.S., and Johnson, J.M. (2005). Microarray analysis shows that some microRNAs downregulate large numbers of target mRNAs. Nature *433*, 769–773.
14. Ma, H., Wu, Y., Dang, Y., Choi, J.-G., Zhang, J., and Wu, H. (2014). Pol III Promoters to Express Small RNAs: Delineation of Transcription Initiation. Mol. Ther. Nucleic Acids *3*, e161.
15. Gu, S., Jin, L., Zhang, Y., Huang, Y., Zhang, F., Valdmanis, P.N., and Kay, M.A. (2012). The loop position of shRNAs and pre-miRNAs is critical for the accuracy of dicer processing in vivo. Cell *151*, 900–911.
16. Gruber, A.R., Lorenz, R., Bernhart, S.H., Neuböck, R., and Hofacker, I.L. (2008). The Vienna RNA websuite. Nucleic Acids Res. *36*, W70–W74.
17. Carette, J.E., Graat, H.C.A., Schagen, F.H.E., Abou El Hassan, M.A.I., Gerritsen, W.R., and van Beusechem, V.W. (2005). Replication-dependent transgene expression from a conditionally replicating adenovirus via alternative splicing to a heterologous splice-acceptor site. J. Gene Med. *7*, 1053–1062.
18. Hauptmann, J., Schraivogel, D., Bruckmann, A., Manickavel, S., Jakob, L., Eichner, N., Pfaff, J., Urban, M., Sprunck, S., Hafner, M., et al. (2015). Biochemical isolation of Argonaute protein complexes by Ago-APP. Proc. Natl. Acad. Sci. USA *112*, 11841–11845.
19. Katoh, M., Igarashi, M., Fukuda, H., Nakagama, H., and Katoh, M. (2013). Cancer genetics and genomics of human FOX family genes. Cancer Lett. *328*, 198–206.
20. Datta, J., Kutay, H., Nasser, M.W., Nuovo, G.J., Wang, B., Majumder, S., Liu, C.G., Volinia, S., Croce, C.M., Schmittgen, T.D., et al. (2008). Methylation mediated silencing of MicroRNA-1 gene and its role in hepatocellular carcinogenesis. Cancer Res. *68*, 5049–5058.
21. Li, J., Kong, X., Zhang, J., Luo, Q., Li, X., and Fang, L. (2013). MiRNA-26b inhibits proliferation by targeting PTGS2 in breast cancer. Cancer Cell Int. *13*, 7.
22. Han, C., Yu, Z., Duan, Z., and Kan, Q. (2014). Role of microRNA-1 in human cancer and its therapeutic potentials. BioMed Res. Int. *2014*, 428371.

23. Zhang, Y.-A., Nemunaitis, J., Samuel, S.K., Chen, P., Shen, Y., and Tong, A.W. (2006). Antitumor activity of an oncolytic adenovirus-delivered oncogene small interfering RNA. Cancer Res. *66*, 9736–9743.

24. Mahoney, D.J., Lefebvre, C., Allan, K., Brun, J., Sanaei, C.A., Baird, S., Pearce, N., Grönberg, S., Wilson, B., Prakesh, M., et al. (2011). Virus-tumor interactome screen reveals ER stress response can reprogram resistant cancers for oncolytic virus-triggered caspase-2 cell death. Cancer Cell *20*, 443–456.

25. Workenhe, S.T., Ketela, T., Moffat, J., Cuddington, B.P., and Mossman, K.L. (2016). Genome-wide lentiviral shRNA screen identifies serine/arginine-rich splicing factor 2 as a determinant of oncolytic virus activity in breast cancer cells. Oncogene *35*, 2465–2474.

26. Rovira-Rigau, M., Raimondi, G., Marín, M.Á., Gironella, M., Alemany, R., and Fillat, C. (2019). Bioselection Reveals miR-99b and miR-485 as Enhancers of Adenoviral Oncolysis in Pancreatic Cancer. Mol. Ther. *27*, 230–243.

27. Teferi, W.M., Dodd, K., Maranchuk, R., Favis, N., and Evans, D.H. (2013). A whole-genome RNA interference screen for human cell factors affecting myxoma virus replication. J. Virol. *87*, 4623–4641.

28. Zheng, J.N., Pei, D.S., Sun, F.H., Zhang, B.F., Liu, X.Y., Gu, J.F., Liu, Y.H., Hu, X.L., Mao, L.J., Wen, R.M., et al. (2009). Inhibition of renal cancer cell growth by oncolytic adenovirus armed short hairpin RNA targeting hTERT gene. Cancer Biol. Ther. *8*, 84–91.

29. Höti, N., Chowdhury, W.H., Mustafa, S., Ribas, J., Castanares, M., Johnson, T., Liu, M., Lupold, S.E., and Rodriguez, R. (2010). Armoring CRAds with p21/Waf-1 shRNAs: the next generation of oncolytic adenoviruses. Cancer Gene Ther. *17*, 585–597.

30. Fan, J., Feng, Y., Zhang, R., Zhang, W., Shu, Y., Zeng, Z., et al. (2019). A simplified system for the effective expression and delivery of functional mature microRNAs in mammalian cells. Cancer Gene Ther. *27*, 424–437.

31. Treiber, T., Treiber, N., and Meister, G. (2019). Regulation of microRNA biogenesis and its crosstalk with other cellular pathways. Nat. Rev. Mol. Cell Biol. *20*, 5–20.

32. Lau, P.-W., and MacRae, I.J. (2009). The molecular machines that mediate microRNA maturation. J. Cell. Mol. Med. *13*, 54–60.

33. Conrad, T., Marsico, A., Gehre, M., and Ørom, U.A. (2014). Microprocessor activity controls differential miRNA biogenesis In Vivo. Cell Rep. *9*, 542–554.

34. Suzuki, K., Alemany, R., Yamamoto, M., and Curiel, D.T. (2002). The presence of the adenovirus E3 region improves the oncolytic potency of conditionally replicative adenoviruses. Clin. Cancer Res. *8*, 3348–3359.

35. Kim, Y.-K., Kim, B., and Kim, V.N. (2016). Re-evaluation of the roles of DROSHA, Export in 5, and DICER in microRNA biogenesis. Proc. Natl. Acad. Sci. USA *113*, E1881–E1889.

36. Dong, W., van Ginkel, J.-W.H., Au, K.Y., Alemany, R., Meulenberg, J.J.M., and van Beusechem, V.W. (2014). ORCA-010, a novel potency-enhanced oncolytic adenovirus, exerts strong antitumor activity in preclinical models. Hum. Gene Ther. *25*, 897–904.

37. R Core Team (2018). R: A language and environment for statistical computing. R Foundation for Statistical Computing, http://www.r-project.org/.

OMTO, Volume *19*

Supplemental Information

Expression of Oncolytic Adenovirus-Encoded RNAi Molecules Is Most Effective in a pri-miRNA Precursor Format

Tereza Brachtlova, Jan-Willem van Ginkel, Mark J. Luinenburg, Renée X. de Menezes, Danijela Koppers-Lalic, D. Michiel Pegtel, Wenliang Dong, Tanja D. de Gruijl, and Victor W. van Beusechem

Supplemental Table 1: Synthetic DNA sequences encoding miRNA inserts prior to cloning into pSHAG-1. shmiR-1-2 and pre-miR-1-2 formats were obtained as two complementary oligonucleotides with overhangs compatible with BseRI/BamHI-digested pSHAG-1; pri-miR-1-1 and pri-miR-26b sequences were obtained as plasmid constructs with flanking restriction sites (coding sequence is given). Blue: passenger strand; Red: mature strand; **Bold**: RNA polymerase III transcription start nucleotide (G) and termination signal (TTTTT) .

Insert	DNA sequences of different microRNA formats
shmiR-1-2 coding	5'-**G**TTACATACTTCTTTACATTCAATAGAAGCTTGTATGGAATGTAAAGAAGTATGTAAC**TTTTT**-<br>3'
shmiR-1-2 complementary	5'-GATCAAAAAGTTACATACTTCTTTACATTCCATACAAGCTTCTATTGAATGTAAAGAAGTAT<br>GTAACG-3'
pre-miR-1-2 coding	5'-**G**GTACATACTTCTTTATGTACCCATATGAACATACAATGCTATGGAATGTAAAGAAGTATGTAT<br>C**TTTTT**-3'
pre-miR-1-2 complementary	5'-GATCAAAAAGATACATACTTCTTTACATTCCATAGCATTGTATCTTCATATGGGTACATAAAGA<br>AGTATGTACCG-3'
pri-miR-1-1	5'-*GAGGAG*GGACTCTACG**G**CAGTAGACTCCAGGGAAGAAGTTACACTGCCTCTGAGCTGCCTTC<br>TCTACATCGCAGTGGGGTCAGCTTCTACCGGGGCGGCGTCCCGGGGTCTTGGAACTGCATGCAGA<br>CTGCCTGCTTGGGAAACATACTTCTTTATATGCCCATATGGACCTGCTAAGCTATGGAATGTAAAG<br>AAGTATGTATCTCAGGCCGGGACCTCTCTCGCCGCACTGAGGGGCACTCCACACCACGGGGGCCG<br>CCGCGGCCGACAGCCAGCCAGGCGGCTGTGGTGGGGGTGAGGCTGGGGCGGCAGGCTGCCTGGG<br>CG**TTTTT**T*GGATCC*-3'
pri-miR-26b	5'-*GCAATG*CG**G**GTGTGAGACTTGTCCCAAAGTCACACAGAACCTCAAGGGCTTGTGCTGACTC<br>CAAGCCTGCAGAGTGGGCTCCTCCTCTAGGCTCCCCCGTGCTGTGCTCCCTCGCCCCACCCTGCCC<br>GGGACCCAGTTCAAGTAATTCAGGATAGGTTGTGTGCTGTCCAGCCTGTTCTCCATTACTTGGCTC<br>GGGGACCGGTGCCCTGCAGCCTTGGGGTGAGGGGGCTGCCCCTGGATTCCTGCACTAGGCTGAGG<br>TTGAGGCAGGGGAAGGGATTGGGAATTAGGGACCTCGTGAGGTAGGACTGGCCAGTGGAGTGGA<br>**TTTTT***GGATCC*-3'

Supplemental Table 2: Virus titers determined as infectious units per ml (IU/ml).

Virus name	Production cell line	Determined titer (IU/ml)
AdΔ24E3-U6	A549	1.1 E+10
AdΔ24E3-U6.shmiR-1	A549	1.6 E+10
AdΔ24E3-U6.pre-miR-1	A549	1.4 E+10
AdΔ24E3-U6.pri-miR-1	A549	1.9 E+10
AdΔ24E3-U6.pri-miR-26b	A549	6.1 E+11
Long-term propagation AdΔ24E3-U6	HCT116	3.4 E+5
Long-term propagation AdΔ24E3-U6.primiR-1	HCT116	3.6 E+5

Supplemental Table 3: Primer sequences used for reverse transcription and qPCR.

Primer name	Primer sequence (5'→3')
PTGS2 forward	CCT GTG CCT GAT GAT TGC
PTGS2 reverse	CTG ATG CGT GAA GTG CTG
MET forward	AAA TAC GGT CCT ATG GCT GGT GGC
MET reverse	TGA AAT GGT TTG GGC TGG GG
FoxP1 forward	GCT TTT TAT GGC TGT GAG ACA CG
FoxP1 reverse	TTC TGG ATG GCT GAA CCG TTA C
U6 primer for RT	GTC ATC CTT GCG CAG G
U6 forward	CGC TTC GGC AGC ACA TAT AC
U6 reverse	AGG GGC CAT GCT AAT CTT CT
SL-miR-26b mature strand for RT	GTC GTA TCC AGT GCA GGG TCC GAG GTA TTC GCA CTG GAT ACG ACA CCT AT
miR-26b mature strand forward	GGG CCC GCT TCA AGT AAT TCA GGA T
SL-miR-26b passenger for RT	GTC GTA TCC AGT GCA GGG TCC GAG GTA TTC GCA CTG GAT ACG ACA GCC AA
miR-26b passenger strand forward	GCC CGC CCT GTT CTC CAT TAC TT
SL-universal reverse	CCA GTG CAG GGT CCG AGG TA
Packaging domain forward	GGA AGT GAC AAT TTT CGC GC
Packaging domain reverse	CCC GCG GCC CTA GAC AAA TAT

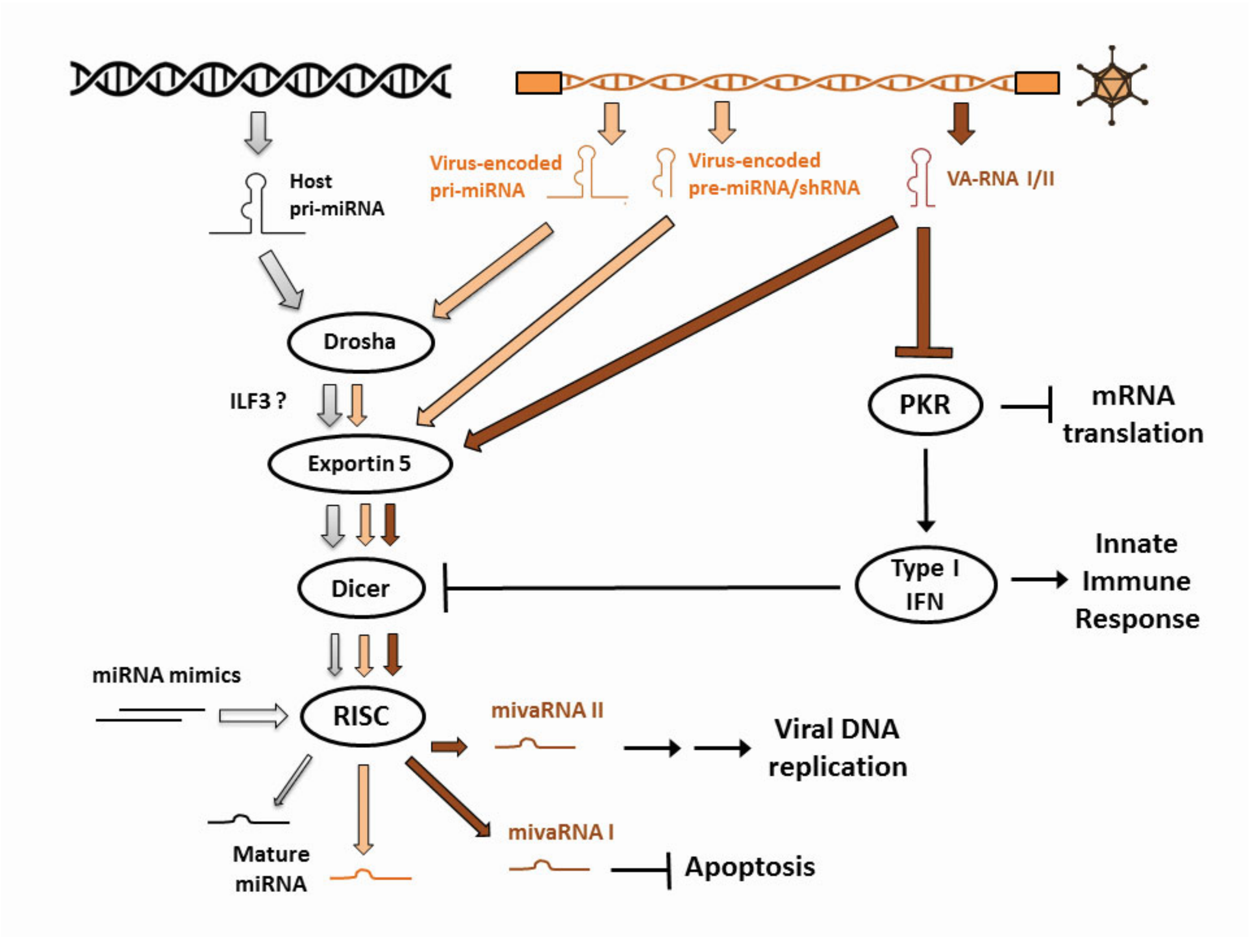

Supplemental Figure 1: Schematic microRNA processing of host-encoded endogenous miRNAs, adenovirus-associated RNAs and virus-encoded exogenous miRNAs.

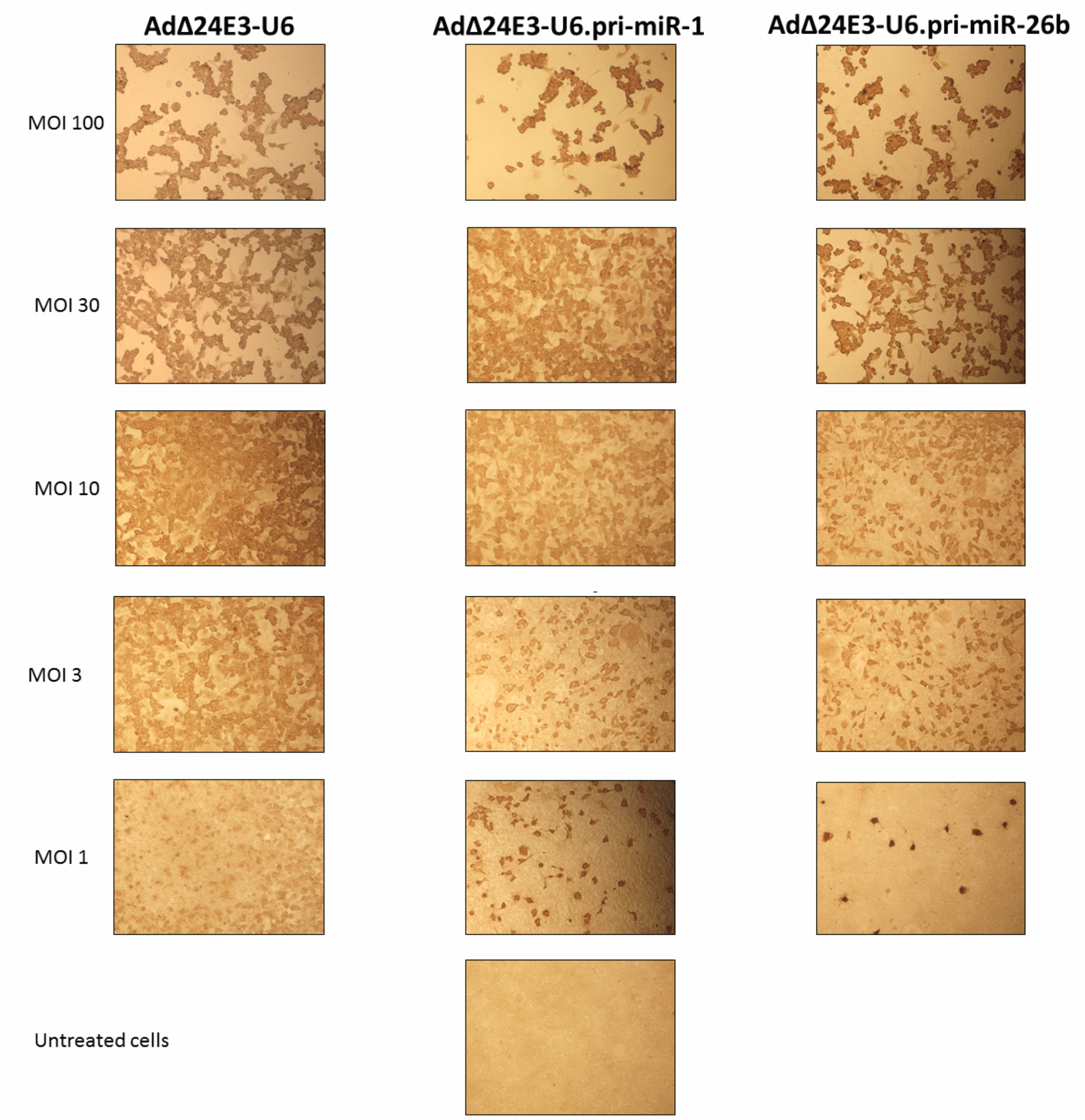

Supplemental Figure 2: Immunocytochemical staining of adenovirus hexon protein in 911 cells infected with AdΔ24E3-U6, AdΔ24E3-U6.pri-miR-1 or AdΔ24E3-U6.pri-miR-26b at various multiplicities of infection. At MOI-100, near complete infection is seen with all tested viruses.

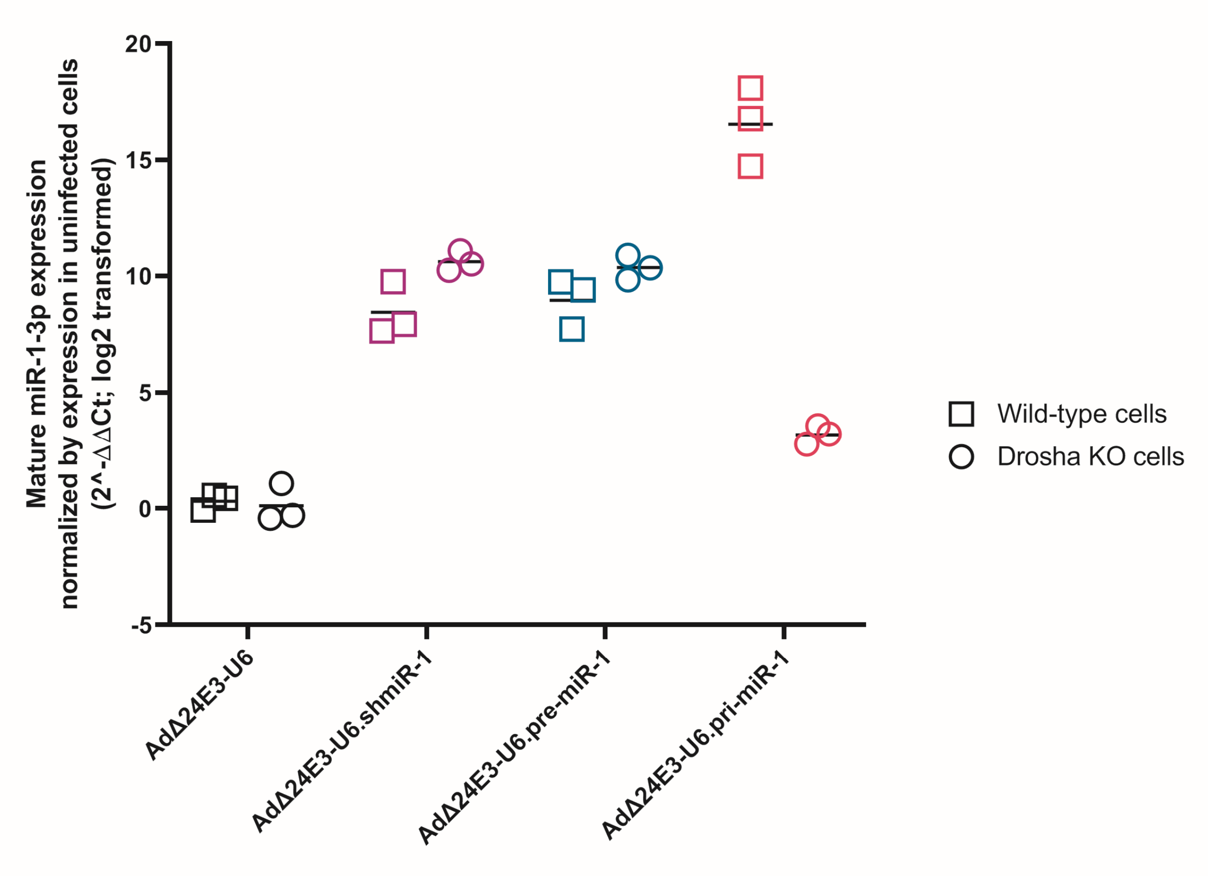

Supplemental Figure 3: The individual measurements in HCT116 wild type cells and *Drosha* knockout (KO) cells after log transformation for correction of skewed data distribution. Each dot represents the mean of one experiment (measured in technical duplicates). Three individual experiments were done.

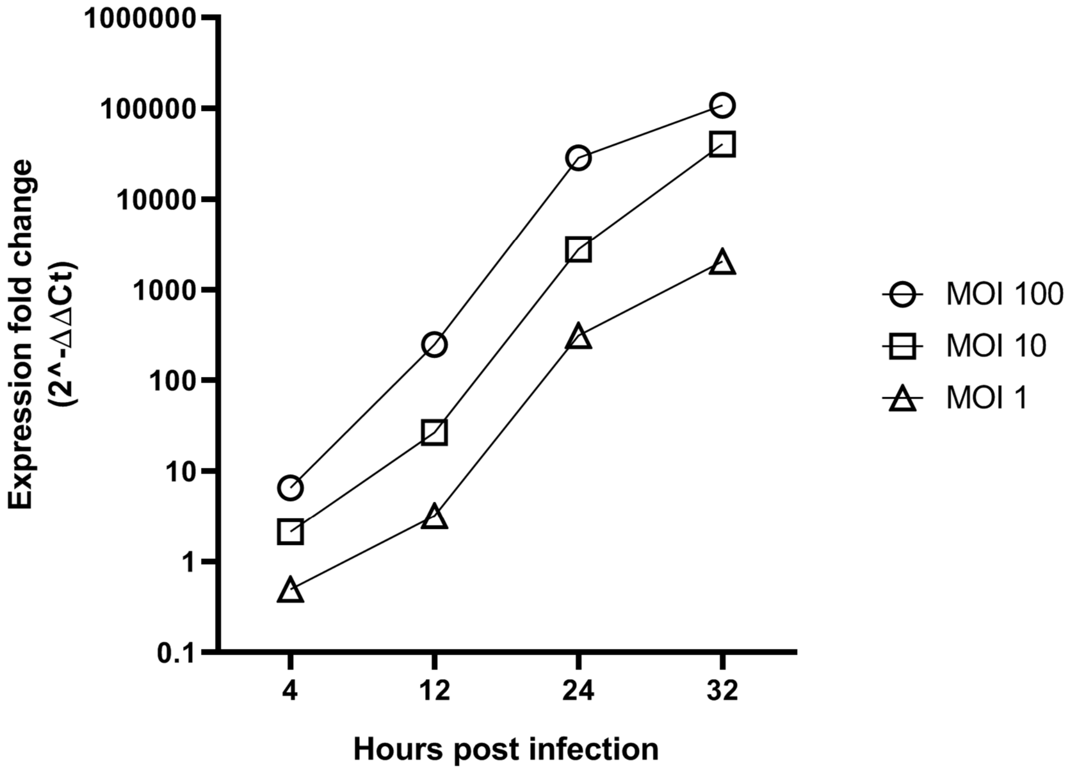

Supplemental Figure 4: Mature miR-1-3p levels in A549 cells infected with AdD24E3-U6.pri-miR-1 at the indicated MOI.

A

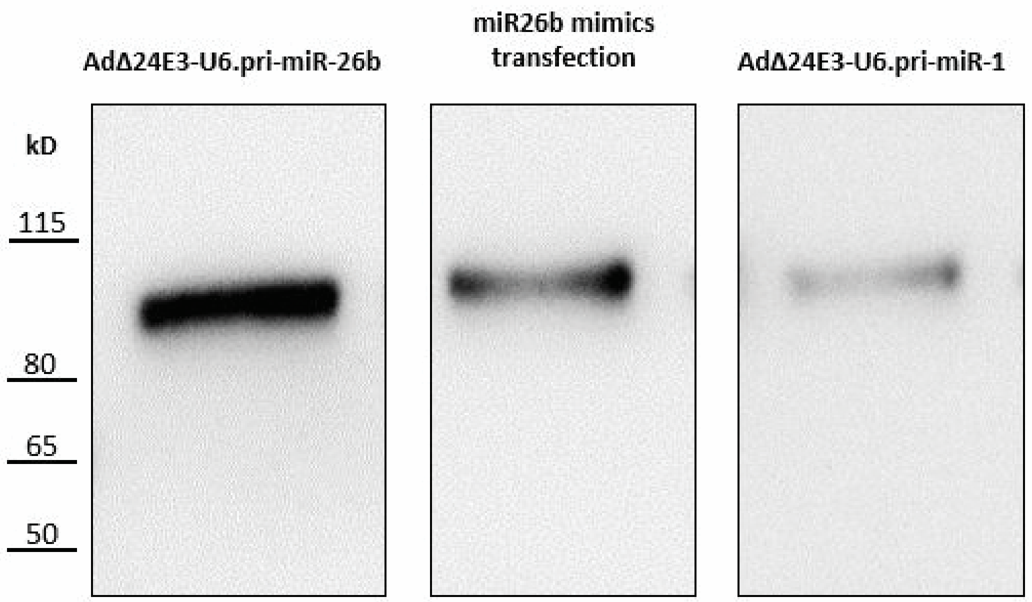

B

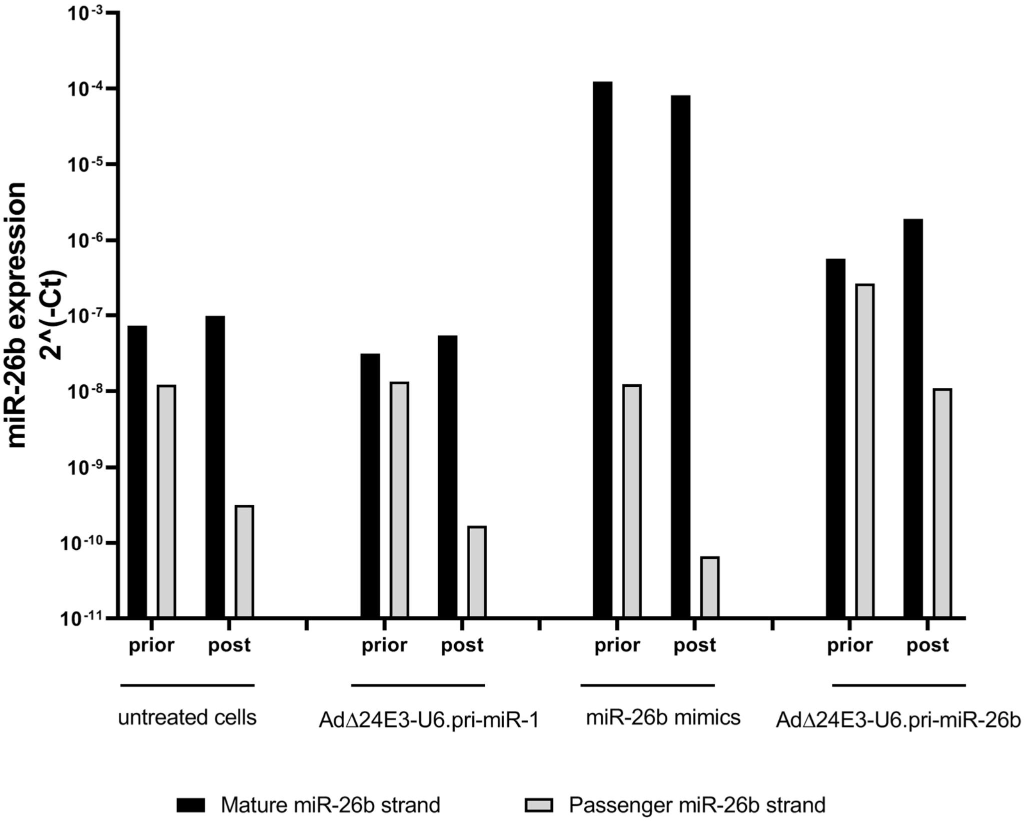

Supplemental Figure 5: Association of miR-26b mature and passenger strands to Argonaute RISC component (AGO) in HCT116 cells. (A) Pulldown of AGO from AdΔ24E3-U6.pri-miR-26b-infected cells, miR-26b mimics-transfected cells and AdΔ24E3-U6.pri-miR-1-infected cells. (B) Levels of mature miR-26b-5p and passenger miR-26b-3p in total cellular RNA (prior pulldown) and AGO-bound RNA (post pulldown). AGO-bound miR-26b was corrected for unspecific binding to beads (negligible). Data are normalized to the same amount input RNA prior to pulldown.
